# Psycho-social factors associated with climate distress, hope and behavioural intentions in young UK residents

**DOI:** 10.1371/journal.pgph.0001938

**Published:** 2023-08-23

**Authors:** Ans Vercammen, Tassia Oswald, Emma Lawrance

**Affiliations:** 1 The School of Communication and Arts, The University of Queensland, St Lucia, QLD, Australia; 2 The Centre for Environmental Policy, Imperial College London, London, United Kingdom; 3 Department of Psychological Medicine, Kings College London, London, United Kingdom; 4 The Institute of Global Health Innovation, Imperial College London, London, United Kingdom; 5 The Grantham Institute for Climate Change and the Environment, Imperial College London, London, United Kingdom; 6 Mental Health Innovations, London, United Kingdom; Nepal Health Research Council, NEPAL

## Abstract

Although the UK has been relatively spared significant geophysical impacts of climate change, many people, youth in particular, are increasingly worried about climate change. The psychological distress associated with the (perceived) threat of climate change has been linked to poorer mental wellbeing but can also promote adaptive responses such as engagement in pro-environmental behaviour. In this mixed methods study, we delve deeper into the experience of ‘climate distress’ among UK residents aged 16–24 (N = 539). We conducted an online survey assessing general mental health, subjective wellbeing, and climate distress with existing scales. We also included novel questions assessing positive and negative life impacts of climate change, open-ended questions on aspirations and priorities for the future, and engagement in pro-environmental and climate actions. Our findings indicate that mental health factors may contribute to vulnerability to climate distress. Predictably, socio-psychological responses to climate change (i.e., frustration over inaction, lack of control, and shame or guilt about one’s own contributions) were linked to higher scores on the climate distress scale. Negatively appraised climate change-related events (i.e., seeing an environment they care about change for the worse) were associated with higher climate distress. Individuals with high climate distress (10.1% of our sample) reported worrying about the impact of climate change on *their own future* more frequently than any other topic surveyed (including personal finance, career, relationships, politics). Both positive (hope/interest) and negative (anger/frustration) emotions inspired action-taking, especially climate activism, which was negatively predicted by guilt/shame and sadness/fear. Private-sphere pro-environmental actions appeared less driven by strong emotions. Overall, our findings present a more nuanced picture of climate distress in terms of emotional responses, behaviour, and mental health. Longitudinal research is urgently needed to understand how distress may change over time, and the conditions that lead to adaptive and maladaptive outcomes.

## 1. Introduction

There is a growing recognition that climate change is affecting human health and wellbeing through a myriad of pathways, including indirect psychological impacts linked to people’s perceptions of the climate crisis [1, 2]. In its most recent assessment report on climate impacts, adaptation, and vulnerability, the IPCC Working Group II has for the first time explicitly highlighted the current mental health challenges of climate change and warns this is only likely to increase [3]. While this projected increase in mental health burden is thought to arise in large part through direct climate impacts (e.g, more people experiencing psychological trauma related to extreme weather events), indirect impacts should not be trivialised, as evident from reports from mental health professionals who support a growing number of clients struggling with their thoughts and feelings around climate change [4–6]. Among other subgroups, young people are disproportionately burdened by climate change. For instance, under current policies, individuals born in 2020 are two to seven times more likely to experience extreme weather events, particularly heatwaves, compared to people born in 1960 [7]. The fact that young people have fewer opportunities for their voices to be heard, or to participate in mitigation and adaptation actions (e.g., if they are not of voting age, or lack decision-making power over domestic pro-environmental actions) can further exacerbate their worry and distress. Even in the absence of personal experience of direct climate impacts, climate change represents a wider existential threat. In their survey of over 10,000 young people globally, Hickman et al. [8] found that over 45% reported their feelings about climate change negatively affected their daily life and functioning. A high proportion (75%) thought the future was “frightening”. This indicates that climate anxiety, a term most commonly used to describe the “heightened emotional, mental or somatic distress in response to dangerous changes in the climate system” [9] is prevalent among this particular demographic. Our own work comparing the psychological impact of the COVID-19 pandemic and climate crisis on young UK residents revealed that even in the context of a global health emergency, young UK residents’ distress about climate change was more pronounced [10].

While there is some debate over definitions of climate-related psychological distress, anxiety responses are a fundamental psychological process that typically serve an adaptive purpose [11], allowing us to prepare proportionate reactions to current and future threats [12]. In line with this interpretation, Reser et al. [13] found that climate distress was the strongest predictor of psychological adaptation to climate change, and that the latter also strongly mediated the relationship between distress and behavioural engagement. While some have warned that climate anxiety may be linked to poorer mental health outcomes, and negatively affect day-to-day functioning [14, 15], other findings suggest a certain degree of anxiety or distress is appropriate and not pathological [16–18]. Climate anxiety can manifest itself also as a practical anxiety, leading to information-seeking and behavioural adaptations [19].

One’s ability to manage or respond to the existential threat of climate change is linked to one’s psychosocial resources, coping skills, and agency to address and mitigate these stressors [20]. This must be contextualised within the wider set of challenges young people contend with, including developmental and life-stage concerns and broader economic, political, and social conditions. Yet research on how young people are coping with climate-related distress is still relatively scarce (but see the work of Ojala, e.g., [21]), and even less is known about the role climate-related worries play in young people’s aspirations. Understanding the conditions under which young people can channel that concern and worry into adaptive actions is crucial to shape appropriate interventions and support. Emerging evidence suggests that collective climate activism can enhance young people’s hope and provide connection to community, both of which can promote personal resilience [22–25]. Hope, as conceptualised in psychology, comprises goals (a desirable outcome), pathway thinking (the ability to generate a route to achieving the goal) and agency (motivation and belief in one’s ability to use the pathway; [26, 27]). In addition to these cognitive elements, different theories recognise the affective dimensions of hope [28]. In this sense, hope is a feeling of possibility, the belief that a problem could be resolved, or conditions could change for the better. The emotion of hope appears to play a primary role in keeping a person engaged with a future, uncertain outcome [29]. In the context of the climate crisis, promoting a hopeful outlook may have dual benefits. First, there is clear evidence that hopeful people feel better, manage stress better, live longer and show more creativity and mental flexibility (for a review, see [30]), all of which are arguably crucial components in promoting wellbeing among young people as they navigate the climate crisis. Second, hope is more likely to inspire action-taking. Especially when it is based on positive re-appraisal, trust in other actors, and trust in one’s own ability to have a positive influence, active hope is a unique motivational force for engaging in pro-environmental behaviours. In contrast, denialist perspectives may inspire a false or passive kind of hope that is linked to disengagement [31, 32]. To understand what gives young people active hope, linked to self-efficacy and action-taking may therefore provide important context to their experience of climate distress.

The research we report here is part of a larger study that was designed to investigate young people’s psychological responses, mental health, agency, behavioural engagement and their visions for the future in the context of co-occurring global crises; the COVID-19 pandemic and climate change [10]. In this paper, we focus specifically on the construct of ‘climate distress’ and what that means for young people in the UK, who–to date–have limited direct experience with climate change impacts. While some psychological impacts may be universal (e.g., high rates of climate ‘worry’ have been noted globally [8]), the social, economic and cultural context provides a unique lens through which climate distress is viewed and experienced. Our aim was to explore the psychological correlates of climate distress using an online survey that was co-designed with young people to ensure that we were able to capture authentic and relevant experiences. Our quantitative approach included validated psychometric scales to allow us to relate our findings to previous observations reported in the growing climate psychology literature. In addition, we solicited and qualitatively analysed free-text comments in an effort to capture novel ideas and relevant contextual dimensions to respondents’ climate change experiences and perceptions.

Our overarching research question was: “If young UK residents experience ‘climate distress’, who is more likely to be affected and how does it present itself”? More specifically, we aimed to address the following specific questions:

1. Is the experience of ‘climate distress’ associated with individual demographic and mental health or wellbeing factors?
2. To what extent is the experience of ‘climate distress’ associated with perceived positive and negative climate impacts?
3. Is the experience of ‘climate distress’ associated with a specific pattern of emotions?
4. To what extent is the experience of ‘climate distress’, and specific climate-related emotions associated with action-taking (e.g., engagement in activism or pro-environmental behaviours)?
5. Does ‘climate distress’ relate to young people’s hopes and worries for their future?

## 2. Materials and methods

The data reported here were collected as part of a larger project comparing psychological responses to the COVID-19 pandemic and climate change in young people resident in the UK (data are available from: https://osf.io/3uztb). For the purposes of the current research, we focused only on the questions relating to climate change, allowing for a more comprehensive analysis of the factors associated with the experience of climate distress, and young people’s hopes for the future.

### 2.1. Ethics statement

The study was reviewed and received approval from the Imperial College London Science, Engineering, & Technology Research Ethics Committee (SETREC; approval number 20IC6060).

### 2.2. Recruitment and sampling strategy

Our initial approach was to reach out to diverse groups of young people via participation calls on websites, existing mailing lists, and social media (Twitter, Instagram, Facebook; for a copy of the study advert, see S1 Text). We specifically targeted young people’s networks (e.g., GenerationR, Talklife, university student societies, youth interest groups), youth-focused charities (e.g., Leaders Unlocked, YoungMinds, Shout, The Mix), and asked other organisations within the researchers’ professional networks to distribute the study adverts. We were deliberate in approaching a broad range of interest groups to not limit recruitment to those already engaged in climate-related activities, and the advert used broadly referred to young people’s feelings in a ‘changing world’, rather than feelings about climate change explicitly. We also asked members of our Young Persons Advisory Group (YPAG), and those who had expressed interest in joining the YPAG, to share the survey within their personal networks. This snowball sampling technique was employed to improve distribution of the survey to a wider public. Our study website also featured a direct link to the online survey. Finally, we also distributed the survey using a paid survey panel (Prolific). This service has a broad reach across the UK, but surveys are completed on a first-come, first-served basis until the sampling quota is reached. While we made efforts to recruit widely, the result is a convenience sample that is not entirely representative of the UK population.

### 2.3. Survey design and distribution

The survey was designed and tested in May-July 2020 and distributed via the Qualtrics survey platform between August 5-October 26, 2020. Prolific respondents were awarded £4 upon completion; respondents in the broader community sample entered a draw to win £100 of shopping vouchers. All respondents provided prior informed consent by ticking the consent box in the first page of the online survey (Following UK specific social research guidelines (e.g. https://the-sra.org.uk) and with approval of the Imperial College London Science, Engineering, & Technology Research Ethics Committee, we considered all participants aged 16 years or above to have capacity to consent and no further parental or guardian consent was obtained). Participants could exit the survey at any time and skip any questions.

The questionnaire included questions and scales adopted from previous studies on climate impacts, or adapted from those developed for impacts of COVID-19 pandemic on young people, as well as questions designed specifically for this study. An initial version of the survey was piloted with our Young Persons’ Advisory Group (YPAG). The YPAG provided comment on the user-friendliness of the survey, the phrasing of the questions and answer formats, as well as contributing more substantively to developing additional items of the climate impacts scales. Full details of the survey are available online (https://osf.io/9ewtn). For the purposes of the current study, we focused on the scales and questions described below (See S1 Table for the exact questions).

#### 2.3.1. Survey measures and outcome variables

*2*.*3*.*1*.*1*. *Mental health and wellbeing*. We used the Patient Health Questionnaire 9 (PHQ-9; [33]), the General Anxiety Disorder Scale 7 (GAD-7; [34]) and the Perceived Stress Scale (PSS; [35]) to assess respondents’ level of depression, anxiety and stress. We also asked respondents whether they had ever received a diagnosis of, or treatment for, a mental health condition, and/or whether they had ever experienced mental ill health (regardless of specific diagnostic criteria or treatment). Standard scoring practices, and–where relevant–the calculation of cut-off scores were followed [33–35].

*2*.*3*.*1*.*2*. *Climate distress and interference with wellbeing*. We used the Climate Distress Scale [13], with one modification—we split one double-barrelled question into two separate questions. The resulting 8 items were rated on a 5-point Likert scale. The total climate distress score was a simple sum of item scores with a possible range of 0–32 (with higher scores indicating higher levels of distress), using mean substitution for missing item values. We employed equivalent cut-off scores as used in the original scale [13] to separate the sample into low, moderate, or highly distressed. We also asked respondents whether their thoughts and feelings about climate change ever interfered with their wellbeing or day-to-day functioning, with a rating on a scale of 0–10 (Not at all–Extremely).

*2*.*3*.*1*.*3*. *Climate impacts and experiences*. Respondents were asked to indicate the three climate impacts that had affected them the most, doing so separately for a list of potential negative impacts and positive impacts. The lists were assembled based on previous studies and through consultation with our YPAG. The negative impacts described (1) *personal and vicarious exposure to specific climate change events* (e.g., seeing upsetting stories in the media, having experienced natural disasters or having a loved one affected by this, local extreme weather and local environmental change), and (2) *broader socio-psychological impacts* (e.g., feeling guilty or remorseful for taking every-day actions that contribute to climate change, getting frustrated over the lack of control over climate change, or the lack of political action on climate change). The latter are conceptually more closely related to the construct of climate distress as it has been explored in the literature to date. After completing the specific impact questions, we asked respondents to rate how much they had been affected overall by the collective negative and collective positive impacts on 5-point Likert scales (not at all–extremely), and how strongly they felt a range of emotions when thinking about climate change. Respondents were provided with a list of 18 emotions based on The Yale Program on Climate Communication [36], and in consultation with our YPAG. To facilitate interpretation, we categorised the climate-related emotions into reactive (‘externalising emotions’: angry, frustrated, disgusted, outraged, disappointed); internally focused (‘internalising emotions’: afraid, sad, anxious, shameful, guilty), goal-oriented (‘approach emotions’: hopeful, interested, engaged, concerned, courageous), and disengaged (‘withdrawal emotions’: helplessness, disconnection, isolation), and we calculated a mean score for each emotion category.

*2*.*3*.*1*.*4*. *Hopes and concerns about the future*. We asked survey respondents to indicate how often they worried about how a range of issues would affect *their own future*. The list of issues was co-created with the YPAG, reflecting common concerns among this demographic. The key difference between the questions on ‘climate distress’ and ‘worry’ (specifically about climate change) is that the former is framed in more general terms and focused on climate impacts in a broad sense, while the latter specifically queries concern about one’s own future prospects in relation to climate change. Respondents were asked to indicate how often they worried about climate change as well as personal finances, work/career, study/school, personal relationships, politics, the economy and COVID-19, each on a 5-point Likert scale (Never–Very often). We also created a relative worry index, which was calculated as the Likert item score for climate divided by the median Likert item score across all other domains. A score greater than 1 would therefore indicate a ‘disproportionate’ degree of climate worry, or a dominance of climate worries over other worries. We also included an open-ended question probing respondents’ hopes for the future, which included “things you wish for, how you see this happening” and/or “things you might fear or that you would like to avoid happening”.

*2*.*3*.*1*.*5*. *Pro-environmental behaviour and climate action*. We asked respondents to what extent they engaged in climate activism *before* the pandemic, because certain activities (e.g., in-person gatherings, school strikes) would have been impacted by COVID-19 restrictions. We also asked how often they “did something for the environment” and provided examples. This was followed by an open-ended question to probe the respondents’ “experience with climate change and the actions you have taken/continue to take to reduce the impact of climate change on your life and/or that of others?”.

*2*.*3*.*1*.*6*. *Demographic questions*. Respondents were asked about their age, gender, ethnicity or cultural background, whether they identified as LGBTQ+, their postcode, and their current living arrangement (e.g., living alone, with parents). To assess their socio-economic position, we used the Family Affluence Scale (FAS-III; [37]).

### 2.4. Analyses

#### 2.4.1. Inclusion and exclusion criteria

To maximise data quality, we applied a series of inclusion criteria to the initial N = 812 survey responses. Ensuring all test responses (N = 2) and automatically identified spam responses (N = 18) were removed left N = 792 potentially valid responses. Of those who started the survey, N = 194 did not click past the consent form. We then applied additional quality control exclusions based on survey progress/missing data (respondents with >50% data missing were excluded), speeding (defined as less than 1/3 of the median response time), and straight-lining behaviour across multiple scales (choosing the same Likert response to all items on a given scale, only applied when half or more of the scales were answered in this way). Application of all three additional criteria resulted in a final N = 539.

#### 2.4.2. Missing values analysis

After applying the above quality checks, we retained a relatively complete dataset. Only one of the study variables (excluding demographic variables) had more than 5% missing values. The question about the extent to which thoughts and feeling about climate change interfered with day-to-day activities had 12% missing responses. However, our analyses were mainly focused on the more reliably measured perceived climate distress rather than functional impairment. As we have reported elsewhere [10], interference with day-to-day activities was (on average) mild, with limited variability in this sample.

#### 2.4.3. Qualitative analysis

We used content analysis, which is a flexible method for analysing text data often applied in mixed-methods health research [38, 39]. It is particularly useful for text data which has been obtained from open-ended survey questions. The purpose of content analysis is to classify large amounts of text into a succinct number of categories that represent similar meanings. Coding categories are derived directly from the text data in an inductive fashion, meaning researchers do not impose preconceived categories or theoretical perspectives on the data. Instead, categories flow from the data and allow new insights to be constructed based on study participants’ perspectives, which are grounded in the data. Results of content analyses are usually presented question by question [40].The content analysis process for the two open-ended questions involved first removing blank cells and responses that did not have meaning or relevance (e.g., “N/A”, “don’t know”). The authors (AV and TKO) read the data in full, to become familiar with the content. Inductive coding of the free-text comments was then conducted by AV for the ‘climate change experience and actions’ question and by TKO for the question about ‘hopes for the future’, using Microsoft Word and Excel. Notes were made of first impressions in the initial coding and peer debriefing was used between the authors to establish credibility of the codes. The codes were assigned to overarching categories, which were further divided into sub-categories through discussion between the authors. Responses that were summative of a category or sub-category were noted and used as example quotes. Concept maps were developed to demonstrate a hierarchy of the categories, sub-categories, and example quotes. The authors (AV and TKO) conducted two rounds of coding checks on a random 15% sub-sample of the data to reflexively improve the analysis, consider inconsistencies, revise the coding frame, and finalise the coding frame before coding all of the data [41]. To determine the coding frame’s external objectivity, a third author who had not been involved in the inductive coding process (EL) independently coded 15% of the data for each question according to the final code frame. Because multiple codes could be assigned to a single element of text and that text can be divided into codable sections in multiple ways, we did not apply inter-rater reliability metrics such as Krippendorff’s Alpha. The percentage agreement for all codes was well above chance level (96.1% for the ‘hope’ question and 96.0% for the ‘climate change experience and actions’ question), indicating very good reliability of the coding frames.

## 3. Results

### 3.1. Sample demographics

Respondents were aged between 16–24 (mean = 21.01, SD = 2.54). The sample consisted of 60.9% women, 31.9% men and 2.2% non-binary individuals; 5% chose not to reveal their gender. 15.8% of the sample identified as LGBTQ+. The majority of the sample was White (68.1%), with 15.8% of Asian descent, 5.3% African/Caribbean, 5.3% mixed-race or belonging to multiple ethnic groups, 0.7% Arab, and 0.2% LatinX; 4.6% did not provide ethnicity data. Based on provided postcode data, the vast majority resided in urban or peri-urban settings (78.8%), while 11.9% lived in a rural area or small town; 9.3% did not provide this information. Most respondents lived with others: family (60.9%), a partner (10.6%) or housemates/friends (15.8%), while only 5.4% lived alone. The remaining respondents specified “other” (1.3%) or chose not to answer (6.1%). The mean Family Affluence Score was 8.23 (SD = 2.27), from possible score range of 0–13, with higher scores indicating a relatively higher socio-economic status. See S1 Fig for a graphical representation of the demographic descriptives.

### 3.2. Climate distress

We found that 36.7% of respondents could be classified as experiencing low levels of climate distress, 53.2% as moderate and 10.1% as high. To examine whether demographic characteristics could explain variation in climate distress, we ran a multinomial logistic regression model with distress level (low, moderate, high) as the outcome variable and age, gender, ethnicity/cultural background, urban vs. rural location, and socio-economic position as predictors. In a second model, we also included whether the respondent had received treatment or been diagnosed with a mental health condition. The inclusion of the latter improved the overall model fit substantially (AIC = 722.732 compared to AIC = 729.716), so we report the full model here. There was a significant overall effect of gender (χ^2^(2) = 7.691, p = .021), socio-economic status (χ^2^(2) = 7.400, p = .025) and mental health status (χ^2^(2) = 14.984, p = .005) on climate distress. Table 1 breaks down the effects for the different distress levels, using low distress as the reference point. A unit of increase on the Family Affluence Scale (indicating a higher socio-economic position) was associated with a significantly higher likelihood of experiencing moderate as opposed to low climate distress (OR = 1.126). Those currently diagnosed and/or receiving treatment for a mental health condition were also significantly more likely to experience moderate as opposed to low climate distress (OR = 1.930) and high as opposed to low climate distress (OR = 3.116). Gender (being male) was associated with a significantly lower likelihood of experiencing high climate distress as opposed to low climate distress (OR = .335). We conducted a similar model with scale scores for depression, anxiety, and stress as indicators of mental wellbeing, rather than focusing on diagnosable or treatable mental health disorders. Only stress significantly explained high climate distress scores (See S2 Table).

**Table 1:** Model results for the multinomial regression on distress categories.

	*B* (SE)	p-value	OR	95% CI for the OR	
				Lower bound	Upper bound
*Moderate vs low climate distress*					
Intercept	1.438 (1.090)	0.187			
Age	-0.076 (.041)	0.068	0.927	0.855	1.006
Gender	-0.314 (.216)	0.146	0.731	0.479	1.116
Ethnicity/cultural background	0.004 (.235)	0.986	1.004	0.634	1.591
Rural vs urban	-0.073 (.312)	0.814	0.929	0.505	1.712
Socio-economic status *	0.119 (.048)	0.012	1.126	1.026	1.236
Mental health = past diagnosis and/or treatment	-0.511 (.326)	0.117	0.600	0.316	1.136
Mental health = current diagnosis and/or treatment *	0.657 (.288)	0.022	1.930	1.098	3.391
Mental health = no history	-				
*High vs low climate distress*					
Intercept	0.746 (1.816)	0.681			
Age	-0.085 (.070)	0.222	0.918	0.800	1.053
Gender *	-1.093 (.432)	0.011	0.335	0.144	0.781
Ethnicity/cultural background	-0.446 (.454)	0.325	0.640	0.263	1.558
Rural vs urban	0.754 (.447)	0.092	2.126	0.885	5.111
Socio-economic status	-0.003 (.077)	0.971	0.997	0.857	1.160
Mental health = past diagnosis and/or treatment	0.606 (.466)	0.193	1.834	0.736	4.571
Mental health = current diagnosis and/or treatment *	1.136 (.441)	0.010	3.116	1.314	7.388
Mental health = no history	-				

Note: B = regression coefficient, SE = standard error, OR = odds ratio, CI = Confidence Interval.

### 3.3. Perceived climate impacts are related to climate distress and emotions

**Fig 1:**
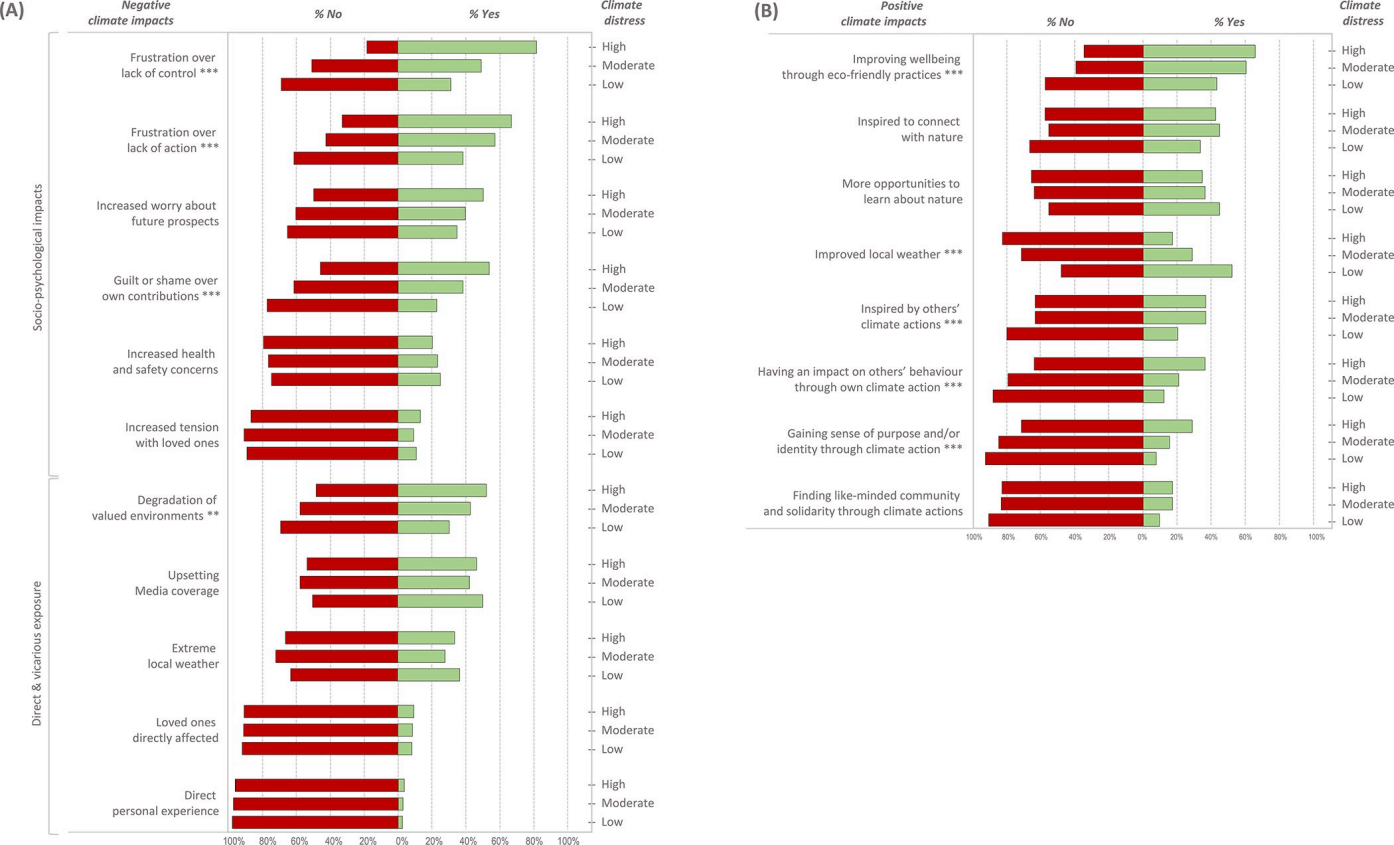
The percentage (of all respondents) who selected specific negative (panel A) and positive (panel B) climate change impacts as being significant for them (as their top three). Significant differences in the proportions of high, moderate, and low distress respondents selecting each impact among their top three are indicated with * p < .05, ** p < .01, *** p < .001.

We conducted Chi-square tests to compare the proportion of respondents who reported experiencing a particular impact across climate distress categories (low-moderate-high; Fig 1). Higher levels of distress were associated with perceived degradation of natural environments that respondents cared about (*χ*^2^(2) = 11.03, p = .004). Climate distress was also associated with certain socio-psychological impacts, namely, frustration over lack of action (*χ*^2^(2) = 22.28, p < .001), lack of control/agency (*χ*^2^(2) = 46.13, p < .001), and feelings of guilt and/or shame over one’s own contributions (*χ*^2^(2) = 22.77, p < .001). The overall perceived severity of negative impacts was strongly associated with greater climate distress (Spearman’s ρ = .566). In terms of positive impacts, we found an association between lower levels of distress and the subjective experience of “better” local weather conditions (*χ*^2^(2) = 35.26, p < .001). Higher distress was associated with positive impacts of taking climate action, namely, the perceived improvement of health and wellbeing from adopting eco-friendly practices (*χ*^2^(2) = 17.05, p < .001), gaining a sense of purpose/identity through climate action (*χ*^2^(2) = 16.97 p < .001), feeling inspired by others’ actions (*χ*^2^(2) = 15.17, p < .001), and being able to influence others’ behaviour through one’s own climate actions (*χ*^2^(2) = 16.49, p < .001). The overall perceived positive impact was not significantly correlated with climate distress (Spearman’s ρ = .016). For the full *χ*^2^ test results, including post-hoc tests pairwise tests, see S3 Table.

We conducted non-parametric correlations to examine the relationship between climate distress and emotions. Overall climate distress was strongly associated with externalising (ρ = .700), internalising (ρ = .747), and approach emotions (ρ = .505), and moderately associated with withdrawal emotions (ρ = .416), suggesting that climate distress is linked to a complex constellation of emotional responses. See S4 Table for the full correlation matrix between climate distress, perceived climate impacts, and each of the climate emotions, and S2 Fig for a graphical comparison of emotion ratings in respondents with low, moderate, and high climate distress. We also explored gender differences in climate emotions, while controlling for the effects of climate distress, noting different patterns in young men compared to young women or gender-diverse individuals; See S3 Fig and S2 Text for details.

### 3.4. Young people’s worries and hopes for the future

We conducted non-parametric correlations, observing that worry frequencies were all positively correlated, with low-moderate effect sizes (Table 2). Climate worry was most strongly correlated with worry about politics.

**Table 2:** Spearman’s ρ correlations among worry dimensions (n = 517); colours indicate small (green), moderate (orange) and large (red) correlations between worry dimensions.

	Climate change	Finances	COVID-19	Study/School	Personal Relationships	Politics	Economy
1.Finances	.139	--					
2.Career	.111	.541	--				
3.COVID-19 pandemic	.294	.357	.341	--			
4.Study/School	.132	.158	.290	.251	--		
5.Personal relationships	.163	.414	.436	.311	.228	--	
6.Politics	.455	.202	.231	.328	.188	.163	--
7.Economy	.272	.448	.414	.393	.179	.232	.415

**Fig 2:**
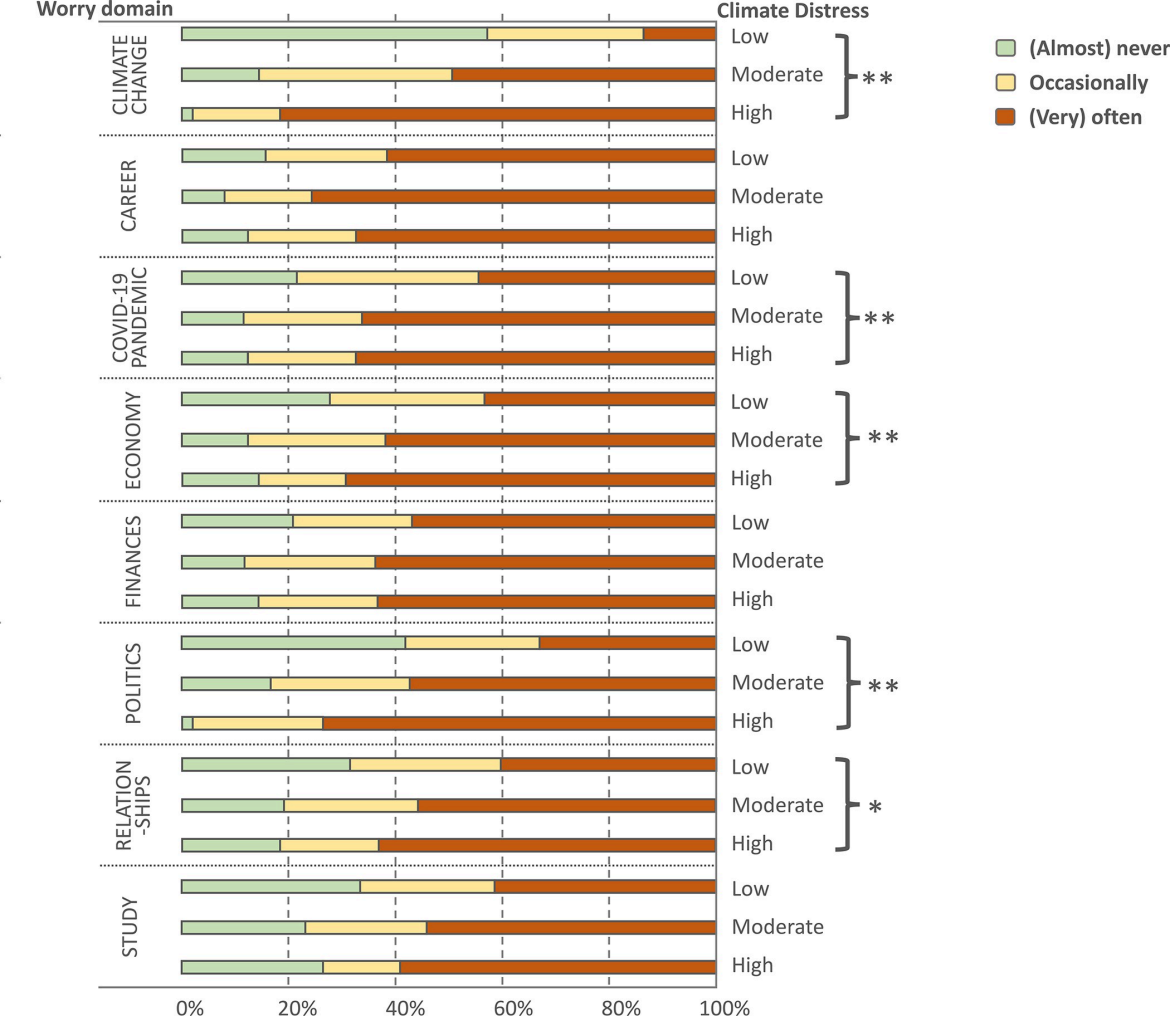
Differences in worry frequency with respect to eight life domains in those with low, moderate and high climate distress. High climate distress is particularly associated with frequent worrying about the impact of climate change and the role of politics on the individual’s future; *p < .05, **p < .01, ***p < .001.

To facilitate analysis and interpretation of the association between climate distress and worry about specific issues, we combined the “Never” and “Seldom” response categories and the “Often” and “Very often” response categories to create a 3-level response variable. We then used Chi-square tests to assess whether there were any associations between climate distress (low, moderate, high) and worry (low, moderate, high) about each of the issues. Among those with high climate distress, climate change was their predominant worry. Higher climate distress was also significantly associated with more frequent worries about the economy, politics, the COVID-19 pandemic, and–to a lesser degree–personal relationships (Fig 2).

The relative worry index was calculated to reflect the psychological burden of climate worries relative to other worries. To explore what explains this relatively high climate change worry, we ran a hierarchical regression model which included demographic predictors in the first step (gender, age, urban/rural, socio-economic status, cultural/ethnic background, overall anxiety level), perceived climate impacts (perceived overall positive and negative impact, total climate distress score) as the second step, and scores on the four climate emotion categories (externalising, internalising, approach and withdrawal) in the third step. The model with just demographic variables was not significantly better than the intercept-only model, (F(6,472) = 1.763, p = .105), but the second (F(9,469) = 14.944, p < .001) and third (F(12,465) = 11.536, p < .001) model were, and each explained additional variance in relative climate worry (Table 3). The full model showed that high climate change worry was significantly linked to greater subjective experiences of negative climate impacts and greater climate distress, but lower generalised anxiety symptoms.

**Table 3:** Coefficient statistics for the hierarchical linear regression models on the relative worry index, which is an indicator of greater climate worries relative to worry about other life domains.

Model		Unstandardized Coefficients		Standardized Coefficients	t-value	p-value	95% CI for B	
		B	SE	Beta			Lower Bound	Upper Bound
1	(Constant)	0.959	0.219		4.385	0.000	0.529	1.388
	Gender	-0.021	0.050	-0.019	-0.418	0.676	-0.119	0.077
	Age	-0.004	0.009	-0.020	-0.424	0.672	-0.022	0.014
	Minority ethnicity	-0.081	0.054	-0.071	-1.515	0.130	-0.186	0.024
	Urban/rural	0.048	0.069	0.032	0.690	0.491	-0.088	0.183
	FAS total score	0.010	0.011	0.043	0.917	0.359	-0.011	0.031
	GAD-7 total score	-0.010	0.004	-0.105	-2.291	0.022	-0.018	-0.001
2	(Constant)	0.622	0.199		3.125	0.002	0.231	1.013
	Gender	-0.089	0.045	-0.083	-1.984	0.048	-0.178	-0.001
	Age	-0.002	0.008	-0.010	-0.234	0.815	-0.018	0.014
	Minority ethnicity	-0.076	0.049	-0.067	-1.558	0.120	-0.173	0.020
	Urban/rural	0.025	0.062	0.017	0.412	0.681	-0.096	0.147
	FAS total score	0.004	0.010	0.017	0.393	0.695	-0.015	0.022
	GAD-7 total score***	-0.021	0.004	-0.227	-5.315	<0.001	-0.029	-0.013
	Negative climate impact***	0.155	0.029	0.269	5.351	<0.001	0.098	0.212
	Positive climate impacts	0.012	0.026	0.019	0.447	0.655	-0.040	0.063
	Climate Distress total score***	0.020	0.004	0.261	5.077	<0.001	0.012	0.028
3	(Constant)	0.515	0.202		2.553	0.011	0.119	0.912
	Gender	-0.072	0.046	-0.067	-1.570	0.117	-0.162	0.018
	Age	-0.002	0.008	-0.010	-0.254	0.800	-0.018	0.014
	Minority ethnicity	-0.079	0.050	-0.069	-1.587	0.113	-0.176	0.019
	Urban/rural	0.033	0.061	0.022	0.541	0.589	-0.087	0.154
	FAS total score	0.000	0.009	0.000	0.008	0.993	-0.019	0.019
	GAD-7 total score***	-0.020	0.004	-0.218	-4.786	<0.001	-0.028	-0.012
	Negative climate impact***	0.143	0.029	0.247	4.905	<0.001	0.085	0.200
	Positive climate impacts	0.006	0.028	0.009	0.201	0.840	-0.049	0.061
	Climate Distress total score*	0.011	0.005	0.145	2.141	0.033	0.001	0.022
	Mean score across (5) externalising emotions	0.078	0.041	0.134	1.923	0.055	-0.002	0.158
	Mean score across (5) internalising emotions	-0.022	0.048	-0.034	-0.457	0.648	-0.116	0.072
	Mean score across (5) approach emotions*	0.106	0.050	0.116	2.142	0.033	0.009	0.204
	Mean score across (3) withdrawal emotions	0.006	0.041	0.007	0.139	0.889	-0.074	0.086

Note: we included N = 479 who provided complete data on all variables, SE = standard error, OR = odds ratio, CI = Confidence Interval.

**Fig 3:**
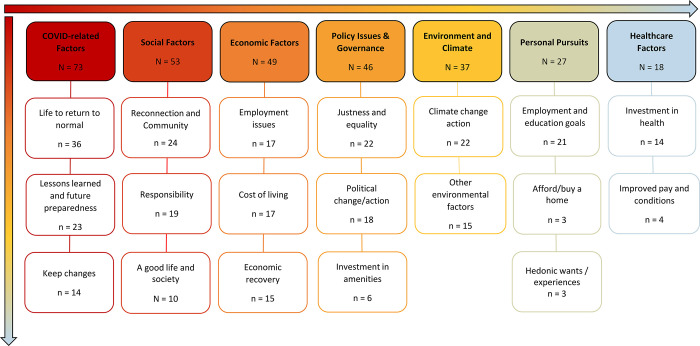
Concept map demonstrating the content analysis of free-text responses pertaining to respondents’ “hopes for the future”. The colour scheme reflects the frequency with which codes indicating a specific theme or subtheme were encountered in the free-text responses (highest = red, lowest = blue).

We recorded 179 free text responses relating to respondents hopes for the future. Of these, 28 were removed because they did not have meaning or relevance (e.g., “no further comment”). The remaining 151 comments were analysed. Seven categories and 19 sub-categories were generated (Fig 3; see S5 Table for additional details). When asked about their hopes for the future, participants most frequently referred to *COVID-Related Factors* (N = 73 responses), followed by *Social Factors* (N = 53), *Economic Factors* (N = 49), *Policy Issues and Governance* (N = 46), *Environment and Climate Change* (N = 37), *Personal Pursuits* (N = 27), and *Healthcare Factors* (N = 18). While most participants indicated their hopes related to these categories, 22% of participants (n = 33) also discussed their fears related to these categories. For COVID-Related Factors, participants most frequently expressed hopes for life to return to normal (n = 36 responses); 83% of these responses were hopeful (e.g., “I wish for things to be how they were before covid”), while 25% also indicated fears that this would not occur (e.g., “Fear that the pandemic could be ongoing for a few years”). Related to Social Factors, participants most commonly expressed a desire for reconnection with their loved ones and to live in a caring community environment (n = 24); 92% of these comments were hopeful (e.g., “I want to see personal relationships get back to the way they were”), while 12% indicated concerns about achieving this (e.g., “I worry that it may be more difficult to make friends and form connections due to social distancing”). For Economic Factors, participants frequently commented on employment issues (n = 17) and the cost of living (n = 17). Regarding employment issues, 65% of comments were expressed as hope (e.g., “I hope that there are more jobs available”), while 35% clearly expressed doubt or concern (e.g., “I fear an even more difficult job market”). Comments about cost of living, were more likely to be expressed in fearful terms (53%, “I fear train ticket prices going up”, rather than hope (47%, e.g., “I hope housing is affordable”). For Policy Issues and Governance, participants most frequently discussed hopes for greater justness and equality in society (n = 22), with 86% of responses indicating hopes (e.g., “End of inequality between the rich getting richer and the poor getting poorer”) and 14% expressing doubts about this (e.g., “I fear … the rich buying up vacant business lots on the cheap and receiving tax cuts while the rest of us go through another 10 years or more of austerity”). Related to Environment and Climate, participants most commonly discussed the need for climate change action, and trust (or lack thereof) in leadership; 86% of comments were hopeful (e.g., “I hope that world leaders will take the issue of climate change more seriously”), while 14% were fearful (e.g., “I fear that there are people in positions of power who still don’t believe in climate change”). The Personal Pursuits most commonly presented by participants were related to employment and education goals (n = 21); 81% of comments indicated hopes about advancing in life (e.g., “I hope to get a job sometime soon”), while 24% also indicated fears about being able to achieve personal goals (“I fear that because of the pandemic I will be unable to get a good job”). Healthcare Factors were typically also linked to awareness of how much the system had been under stress due to the COVID-19 pandemic. Participants most frequently commented on the need for greater investment in health systems (n = 14); 86% of these comments were positively framed (e.g., “I hope that the national health service can recover, and be better funded in future”), and 14% were fearful about the future of health and social care systems (e.g., “I fear that our government will sell out the NHS and fully privatise health care”).

### 3.5. Engagement in climate activism and pro-environmental behaviour

We conducted a binary logistic regression model on self-reported engagement in climate activism (yes/no), with demographic variables as the first set of predictors. In the second step, we included climate distress as an additional predictor, and in the third step we included the four climate emotion categories as additional predictors. All the models significantly predicted engagement in climate activism, and each subsequent step increased the amount of variance explained in the outcome variable. Thus, both climate distress and climate emotions significantly improved the predictive ability of the model (S6 Table). The full model explained a significant amount of variance, χ2(10) = 126.568, p < .001, Nagelkerke R^2^ = .349. Age was the only significant demographic predictor, with older respondents less likely to be engaged in climate activism. Individuals experiencing higher climate distress were more likely to report engaging in climate activism. Finally, stronger externally focused emotions and approach emotions significantly predicted a greater likelihood of engaging in climate activism, while stronger endorsement of withdrawal emotions reduced the likelihood (see Table 4 for model coefficients).

**Table 4:** Individual predictor coefficients for the binary logistic regression model predicting participation in climate activism.

	B	S.E.	Wald	df	p-value	OR	95% C.I.for Exp(B)	
							Lower bound	Upper bound
Constant	-1.630	1.197	1.854	1	.173	.196		
Gender (0 = men; 1 = other)	.320	.302	1.118	1	.290	1.377	.761	2.490
Age**	-.162	.050	10.670	1	.001	.850	.771	.937
Ethnicity/cultural background (0 = white European; 1 = other)	-.259	.311	.695	1	.404	.772	.420	1.419
Geographic location (0 = urban; 1 = rural)	.254	.365	.485	1	.486	1.290	.630	2.640
Socio-economic status	.057	.059	.944	1	.331	1.059	.943	1.189
Climate distress score*	.072	.030	5.961	1	.015	1.075	1.014	1.140
Externalising emotions***	.871	.196	19.662	1	< .001	2.388	1.626	3.510
Internalising emotions	-.246	.215	1.316	1	.251	.782	.513	1.191
Approach emotions***	.719	.182	15.561	1	< .001	2.052	1.436	2.933
Withdrawal emotions**	-.478	.164	8.469	1	.004	.620	.450	.856

Note: B = regression coefficient, SE = standard error, Wald = Wald statistic, df = degrees of freedom, OR = odds ratio (defined as exp(B), CI = Confidence Interval.

A similar series of regression models was formulated to predict how often people engaged in pro-environmental behaviours (never, occasionally, or frequently), based on demographic variables (Step 1), climate distress (Step 2), and climate emotions (Step 3). S7 Table provides full details on model statistics and predictor coefficients at each step. Each model significantly predicted pro-environmental behaviour, and each subsequent step lowered the AIC value, suggesting an increasingly better fit with the inclusion of the additional predictors. The full model explained a significant amount of variance, χ2(10) = 93.327, p < .001, and revealed that more frequent engagement in pro-environmental behaviour was predicted by ethnicity/cultural heritage (White/European heritage compared to minority groups), although the confidence interval was quite wide, indicating uncertainty about the population parameter. Both higher climate distress, and higher scores on approach emotions were linked to more frequent engagement in pro-environmental behaviour (See Table 5).

**Table 5:** Individual predictor coefficients for the ordinal regression model predicting self-reported participation in pro-environmental behaviours.

	B	SE	Wald	df	p-value	95% CI for the OR	
						Lower bound	Upper bound
Gender (0 = men; 1 = other)	.350	.2100	2.777	1	.096	-.062	.761
Age	.071	.0377	3.497	1	.061	-.003	.145
Ethnicity/cultural background (0 = white European; 1 = other)*	.512	.2226	5.285	1	.022	.075	.948
Geographic location (0 = urban; 1 = rural)	.059	.2849	.042	1	.837	-.500	.617
Socio-economic status	.067	.0435	2.405	1	.121	-.018	.153
Climate distress score***	.084	.0227	13.558	1	< .001	.039	.128
Externalising emotions	.156	.1477	1.112	1	.292	-.134	.445
Internalising emotions	-.061	.1651	.137	1	.711	-.385	.263
Approach emotions***	.615	.1413	18.944	1	< .001	.338	.892
Withdrawal emotions	-.107	.1237	.752	1	.386	-.350	.135

Note: B = regression coefficient, SE = standard error, Wald = Wald statistic, df = degrees of freedom, OR = odds ratio (defined as exp(B), C.I. = Confidence Interval.

**Fig 4:**
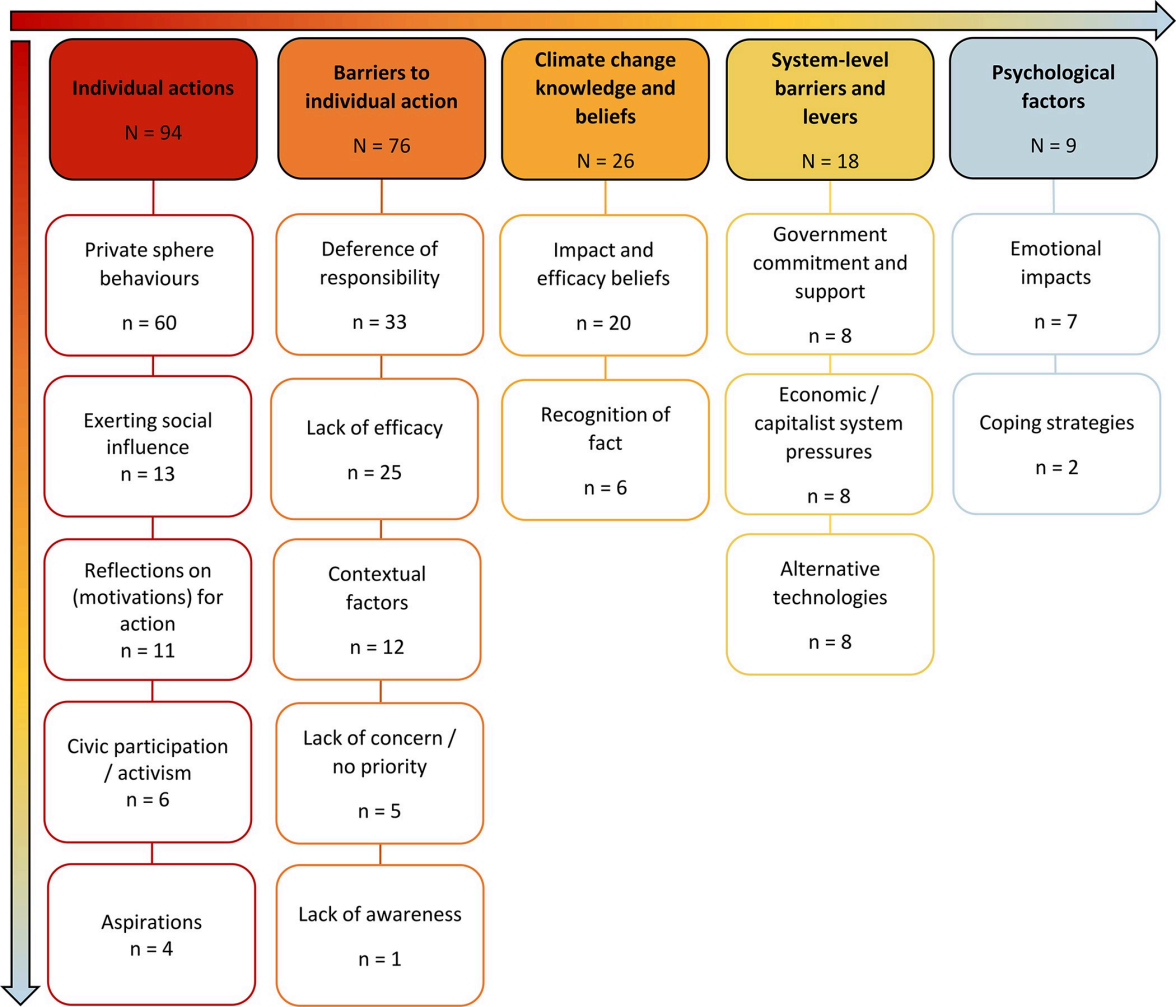
Concept map demonstrating the content analysis of free-text responses pertaining to respondents’ “experience with climate change and the actions they were taking in response to climate change”. The colour scheme reflects the frequency with which codes indicating a specific theme or subtheme were encountered in the free-text responses (highest = red, lowest = blue).

We recorded 187 free text responses relating the respondents’ experience with climate change and climate action. Of these, 65 were removed because they did not have meaning or relevance, or were non-responses (e.g., “no further comment”). The remaining 121 comments were analysed. Five categories and 17 sub-categories were generated (Fig 4; see S8 Table for additional details).

Respondents most commonly referenced *Individual Actions* (n = 94) in response to the question about their engagement with the issue of climate change. This category included reports of engagement in pro-environmental behaviours (e.g., “My friends and I regularly cycle to see each other instead of taking an Uber”). The vast majority referred to typically low-impact every-day activities taking place in the private sphere, such as recycling or reducing single-use plastics. Some responses described respondents’ public actions such as civic participation or political activism, but this was far less commonly reported. The remaining subcategories reflected respondents’ encouragement of others to take up similar behaviours, aspirations towards pro-environmental actions and/or sustainable living (rather than actualised behaviour), and respondents reflecting on the motivations that underpinned these actions. The counterpart to this category was *Barriers to Individual Action* (n = 76), which highlights various reasons or explanations for the lack of individual action. The most common reason was deferring of responsibility to other actors (e.g., “It’s the fat cat corporations that really should be making changes”), followed by the perception that individual actions were simply ineffective or pointless (e.g., “I just don’t think it makes much difference in the grand scheme of it all”). Some noted contextual factors such as a lack of alternatives, or their cost, insufficient knowledge, or–typically perceived in others–unwillingness to make changes or reset priorities. In addition to factors inhibiting change at the individual level, some respondents also identified *System-Level Levers and Barriers* (n = 18), particularly around government commitments and capitalist pressures or economic drivers (e.g., “We need to shift away from consumerism and materialist cultures and think about distributing power and wealth”). Respondents also shared insights into their *Climate Change Knowledge and Beliefs* (n = 26), mainly focusing on the perceived impact of current or future mitigation and adaptation actions (e.g., “I don’t think it’s too late to act, but we have to change things now”). Only a few respondents shared their reflections on the reality of living with climate change. The smallest category mentioned *Psychological Factors* (n = 9). This mainly revolved around emotional responses (e.g., “I try to do my part, but I also feel guilty because I could be doing more”), or on ways in which respondents were attempting to cope with climate change (e.g., “I try not to think about it too much”).

## 4. Discussion

Recent research has demonstrated that climate anxiety is an increasingly common experience, especially among youth [5, 8, 15, 42, 43]. We have previously reported on young people’s psychological responses to climate change and the COVID-19 pandemic, demonstrating that distress about climate change was greater than distress about the pandemic [10]. In the current study, we queried additional data obtained from the extensive online survey we conducted in 2020. This investigation aimed to provide a broader assessment of the factors that contribute to the experience of ‘climate distress’ among this group of young people in the UK. We focused on understanding who is experiencing climate distress, and how this relates to patterns of emotional responses as well as future worries, climate action, and pro-environmental behaviour. To provide richer context to the experience of climate distress, we gathered and analysed free-text responses complementing the quantitative survey questions.

First, a number of demographic factors were significantly associated with climate distress, although they explained only a small amount of variation. We found that moderate climate distress was more likely in individuals in higher socio-economic brackets, and among those currently experiencing a mental health condition. High climate distress was more likely among women and gender-diverse individuals. These findings align with other studies reporting demographic variation in climate distress or related concepts such as eco-anxiety [44–46]. Further investigation into the mechanisms that underpin these individual-level vulnerabilities is crucial. Arguably, priority might be given to further research assessing the possible association between climate distress and mental ill health. While there is a general consensus that climate distress is non-pathological, aspects of the experience intersect with diagnosable anxiety conditions. Further research is needed to clarify the nature and directionality of this effect. Climate distress may be a short-term or long-term risk factor for the development of mental health conditions. On the other hand, pre-existing conditions such as anxiety and depression, or their underlying drivers, may pre-dispose young people to heightened climate distress [47]. Indeed, both may be true, and interactions are plausible. Our findings do not conclusively point to a specific explanation but are consistent with the proposition that climate-related distress may add to the mental health burden in an already vulnerable group of people.

There was no indication that the more highly distressed respondents had experienced more extreme weather impacts (personally or vicariously) or had more exposure to upsetting media coverage. However, they did report being affected by environmental degradation of places they cared about. This was coupled with greater frustration over the perceived lack of action on climate change, a lack of personal agency, concern over one’s future, and feelings of guilt and shame. While this may start to paint a concerning picture of loss and despair, there were notable positive experiences linked to climate distress, too. Highly distressed respondents were more likely to report deriving meaning, fulfilment, and wellbeing benefits from engaging in climate action. Climate distress therefore seems to be a multidimensional experience, a varying constellation of both subjectively positive and negative aspects. We find that in a sample of young people with little or no exposure to physical impacts, distress may be an almost unavoidable correlate of engagement with the issue of climate change, even when the effects are distant in time and space.

In line with this interpretation of climate distress as an integral component of engagement, we found that climate distress significantly explained self-reported action-taking, both in the form of activism and (private) pro-environmental behaviour. While directionality of this relationship cannot be assumed, this finding is consistent with the interpretation that heightened concern for the issue is a motivator for change (e.g., [18]). Activism, a public demonstration of engagement was strongly driven by emotional reactions, over and above the effect of climate distress. Climate-related emotions that are focused outward, with negative valence (e.g., anger, frustration, disappointment) or positive valence (e.g., hope, courage, interest) were associated with higher likelihood of engagement in activism. Emotions that indicated withdrawal from the issue (e.g., helplessness, disconnect) were, perhaps unsurprisingly, associated with a lower likelihood. These findings echo previous observations that anger is a key driver of engagement with the climate crisis, and particularly in collective action [48, 49]. When angered (rather than fearful or saddened), people are more likely to see opportunities for action and engage with determination and courage [50]. While some studies have found that eco-shaming and feelings of guilt [51] can be an impetus for behaviour change, we found no association between these internally focused emotions and behavioural engagement.

Unlike activism, which is by its very nature a collective and public stance, many pro-environmental behaviours take place in the private sphere and are less visible. In line with other research, we found that these kinds of behaviours were less frequently reported by individuals from lower socio-economic and in minority groups [52, 53]. However, when we took into account climate distress, that relationship was no longer significant, suggesting that differences in overall climate-related concern may have been driving the demographic variation in pro-environmental behaviours. These pro-environmental behaviours were associated with positive, goal-oriented emotions (e.g., hope, courage, interest), but none of the other emotion categories. The free-text responses indicated that participants were often quite committed to these private-sphere pro-environmental behaviours, despite questioning the efficacy of these same actions, and shifting responsibility on to other societal actors with more power to effect change. This suggests that the emotional component of hope may play a key role in maintaining pro-environmental behaviour even when agency is limited. Overall, our finding that some emotions like hope and anger are more likely to inspire action-taking than others, and that this differs for behaviour that happens in the public or private sphere, suggests that a more fine-grained conceptualisation of the emotional drivers of environmental and climate action is needed. This echoes a recent call for a deeper understanding of the functional profiles of ecological emotions [19]. More research is also needed to establish the extent to which cultivating these potentially intense emotions in the longer term might affect individual wellbeing [54]. Maintained states of anger can have lasting health consequences [55]. In the current sample, emotional reactions were moderate in severity, but little is known about how and when these mild experiences may tip over into risk factors for poor mental health [47]. Young people experiencing any level of distress may still benefit from tools and techniques that assist them in recognising and leveraging helpful emotions while learning to cope with potentially less adaptive emotions like guilt and shame. Exploration of therapeutic approaches like acceptance and commitment therapy may hold promise here [56].

As mentioned earlier, our qualitative analyses supported the idea that many young people were aware of, and committed to every-day, private-sphere actions. Most respondents focused on relatively low-impact behaviours such as recycling or refusing single-use plastics, although the more impactful option of making environmentally motivated dietary changes was also reasonably common among this demographic. Interestingly, many young people appeared to persist in these private-sphere actions, despite openly questioning their efficacy. Few reported a willingness or ability to make more substantive lifestyle changes, or to put pressure on and influence decision-makers directly. Instead, responsibility was often passed to other actors, chiefly corporates and governments who were perceived to wield more power to drive systemic change. Some respondents specifically expressed dismay at the unsustainable nature of a system driven by profit-making and greed. Our observations echo findings from the 2021 global youth survey which showed widespread dissatisfaction and frustration with government responses to climate change [8]. These observations point to a lack of active hope, which is based on trust in other actors, and trust on one’s own ability to have a positive influence. Active hope is a unique motivational force for engaging in action-taking and is protective of wellbeing in the face of climate concerns [31]. Given the indications that collective action may be protective against adverse mental health outcomes in the context of climate distress [22], it seems obvious that more could be done to empower young people with an appropriate and meaningful outlet for their distress.

To understand climate distress in context, we also queried respondents on other issues that concerned them, specifically in terms of how they might affect *their own future*. Worry about different issues has previously been found to be positively correlated (e.g., climate and pandemic-related worries, see [57]), and this was true in the current sample as well. This indicates that climate worries may be driven by an underlying individual propensity to worry about threats in general. Interestingly, climate worry was most strongly correlated with concern about politics. This may be another indication that threat perceptions about climate change may exacerbate or be exacerbated by a lack of trust in government and decision-makers. When asked to elaborate on their hopes and dreams for the future, some respondents explicitly pointed out the need for more political will and action on key community issues. This included the desire for social justice and equity, as well as the hope that climate action would be taken seriously by those in power. Still, most young people’s immediate, personal worries centred on career and finances rather than climate change. Likewise, the most hopes for the future focused on COVID-19 control, economic recovery and social reconnection, which was to be expected at the height of the pandemic in the UK. This may suggest that for many, concern about climate change remains rather abstract, and not specifically linked to their own lives. It was only among those with particularly high climate distress that the climate crisis came out as their top concern (more than school, work, relationships, politics, the economy etc.). The fact that for 10% of the respondents, concern about climate change was the most frequent cause of worry for their own future, even in a country not yet highly threatened by climate hazards, remains striking. While such worry is not necessarily pathological in nature, there is an urgent need to develop appropriate resources to support young people experiencing high climate distress, to help them navigate their climate concerns alongside other developmental challenges in these formative years.

Before we conclude, several limitations are worth noting. The cross-sectional survey design does not allow us to draw inference about the directionality of the observed relationships between climate distress and the socio-psychological antecedents or consequences. The data also represent a unique snapshot of young people’s thoughts and feelings around climate change taken in the context of a particularly challenging period, i.e. at the height of pandemic restrictions in the UK [10]. This unique set of circumstances will likely have influenced responses, particularly with respect to young people’s hopes for the future. As the UK, and the world emerges from the pandemic and faces other challenges (e.g., economic recession), patterns may again start to shift. Although we employed a broad approach to recruitment through a variety of youth organisations (not those purely focussing on climate) as well as a well-established research panel service, our survey did not reach a representative sample of UK youth. The current sample was more ethnically and culturally diverse than the UK population. We also reached more women and gender-diverse individuals than would be expected in a representative survey. Our convenience sampling method may have led to a potentially biased response pool of young people with a specific interest in climate change and/or (mental) health, as the broader survey also included questions about pandemic impacts. However, it was not our aim to make prevalence estimates about climate distress, but rather to explore the relationships between the experience of climate distress and a range of socio-psychological variables, producing novel insights into potential drivers and consequences of climate distress. There is still an urgent need for large-scale representative surveys, longitudinal studies, and in-depth qualitative work with diverse groups of young people to develop a more profound understanding of the mechanisms that underpin climate-related distress, and how this might interact with societal and geopolitical change.

## 5. Conclusions

It is now well established that the impacts of climate change go beyond geophysical changes. Our findings highlight that–even in the absence of direct climate-related experiences–distress about climate change is affecting young people’s aspirations, breeding distrust and frustration with decision-makers, and potentially curtailing their personal growth. For those experiencing high climate distress, climate change may become their main worry, exacerbating, or creating anxiety disorders. The emotional responses that drive engagement in climate activism appear to have short-term positive impacts such as finding supportive communities and hope in collective action. We do not know whether maintaining climate distress may have negative consequences in the longer term. Further research is urgently required to identify the mechanisms that underpin adaptive and maladaptive pathways associated with climate distress, and to develop age-appropriate, effective support to aid young people to thrive, and to engage safely and effectively in mitigating the climate crisis.

## Supporting information

S1 Fig Demographic descriptives for the sample. (TIF)

S2 Fig Radar graph of emotion ratings for respondents with varying levels of climate distress, visualising the differences in mean scores for externalising (red), internalising (light blue), approach (white) and withdrawal (light pink) climate emotions between respondents with low climate distress, moderate climate distress and high climate distress. The observed pattern suggests that the most pronounced differences between distress groups are found in externalising emotions.
(TIF)

S3 Fig Estimated marginal means of emotion ratings for externalising, internalising, approach and withdrawal emotions in men and women or third gender individuals. Differences between groups are presented at the mean covariate values: GAD-7 total score = 8.01, Climate distress total score = 13.0840.
(TIF)

S1 Table List of survey questions used in the current analyses. The full survey is available from: https://osf.io/9ewtn.
(DOCX)

S2 Table Model results for the multinomial regression on distress categories; with inclusion of depression, anxiety and stress scores as predictors. (DOCX)

S3 Table Chi-square tests assessing the association between distress levels (low/moderate/high) and reporting (yes/no) of specific climate impacts. ***p < .001; **p < .01. *p < .05 (applies to the omnibus test comparing all three levels of distress for presence/absence of each specific impact). In addition, we report follow-up pair-wise tests comparing low-moderate, moderate-high and low-high levels of distress groups on presence/absence of each of the specific impacts.
(DOCX)

S4 Table Correlation matrix between climate distress, overall perceived extent of positive and negative climate impacts and climate-related emotions. (DOCX)

S5 Table Description and frequency of categories and sub-categories for the free-text responses pertaining to respondents’ reported “hopes for the future”. (DOCX)

S6 Table Results of the binary logistic regression model predicting participation in climate activism: Model statistics at each step, model statistics for the comparison with the previous model (Δ Step), and individual predictor coefficients. Note: B = regression coefficient, SE = standard error, Wald = Wald statistic, df = degrees of freedom, OR = odds ratio (defined as exp(b), CI = Confidence Interval.
(DOCX)

S7 Table Results of the ordinal regression models predicting self-reported participation in pro-environmental behaviours: Model statistics at each step, model fit, and individual predictor coefficients. Note: B = regression coefficient, SE = standard error, Wald = Wald statistic, df = degrees of freedom, OR = odds ratio (defined as exp(B), CI = Confidence Interval, AIC = Akaike Information Criterion.
(DOCX)

S8 Table Description and frequency of categories and sub-categories for the free-text responses pertaining to “Climate change experiences and actions”. (DOCX)

S1 Text Example adverts used to recruit participants on social media. (DOCX)

S2 Text Statistical tests comparing reported emotional responses to climate change between genders (men vs other genders). (DOCX)
