# Supplementary figures and images for: Psycho-social factors associated with climate distress, hope and behavioural intentions in young UK residents
Source: PLOS Glob Public Health. 2023 Aug 23;3(8):e0001938. doi: 10.1371/journal.pgph.0001938 (PMC10446227; doi:10.1371/journal.pgph.0001938)

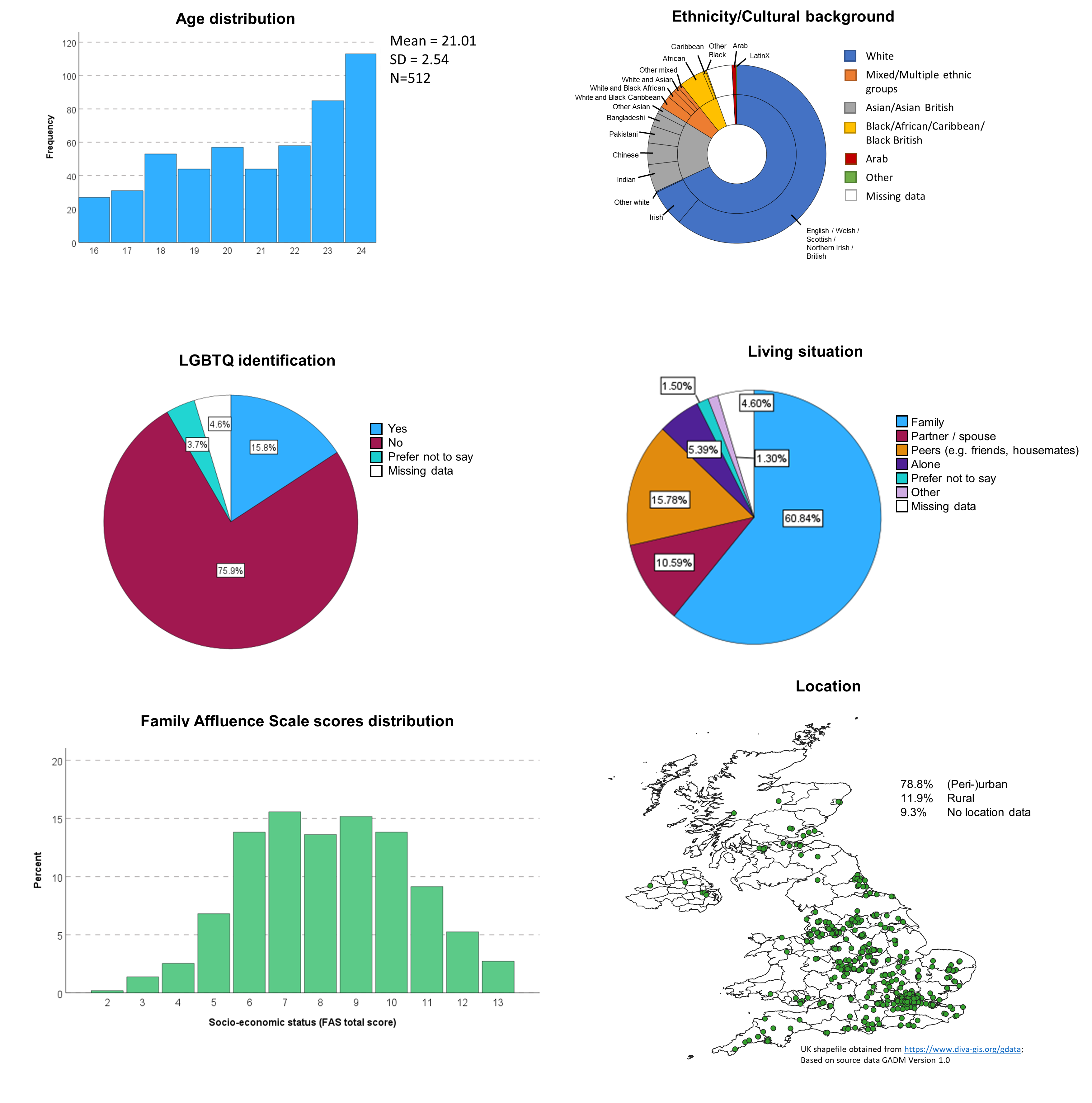

Supplement: S1 Fig — (TIF) [file pgph.0001938.s001.tif]

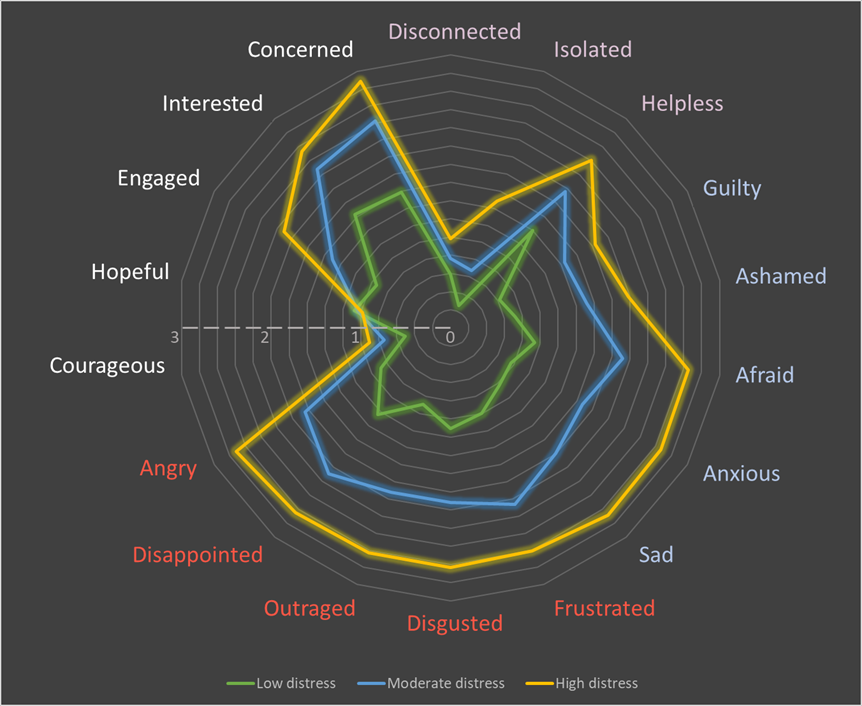

Supplement: S2 Fig — The observed pattern suggests that the most pronounced differences between distress groups are found in externalising emotions. (TIF) [file pgph.0001938.s002.tif]

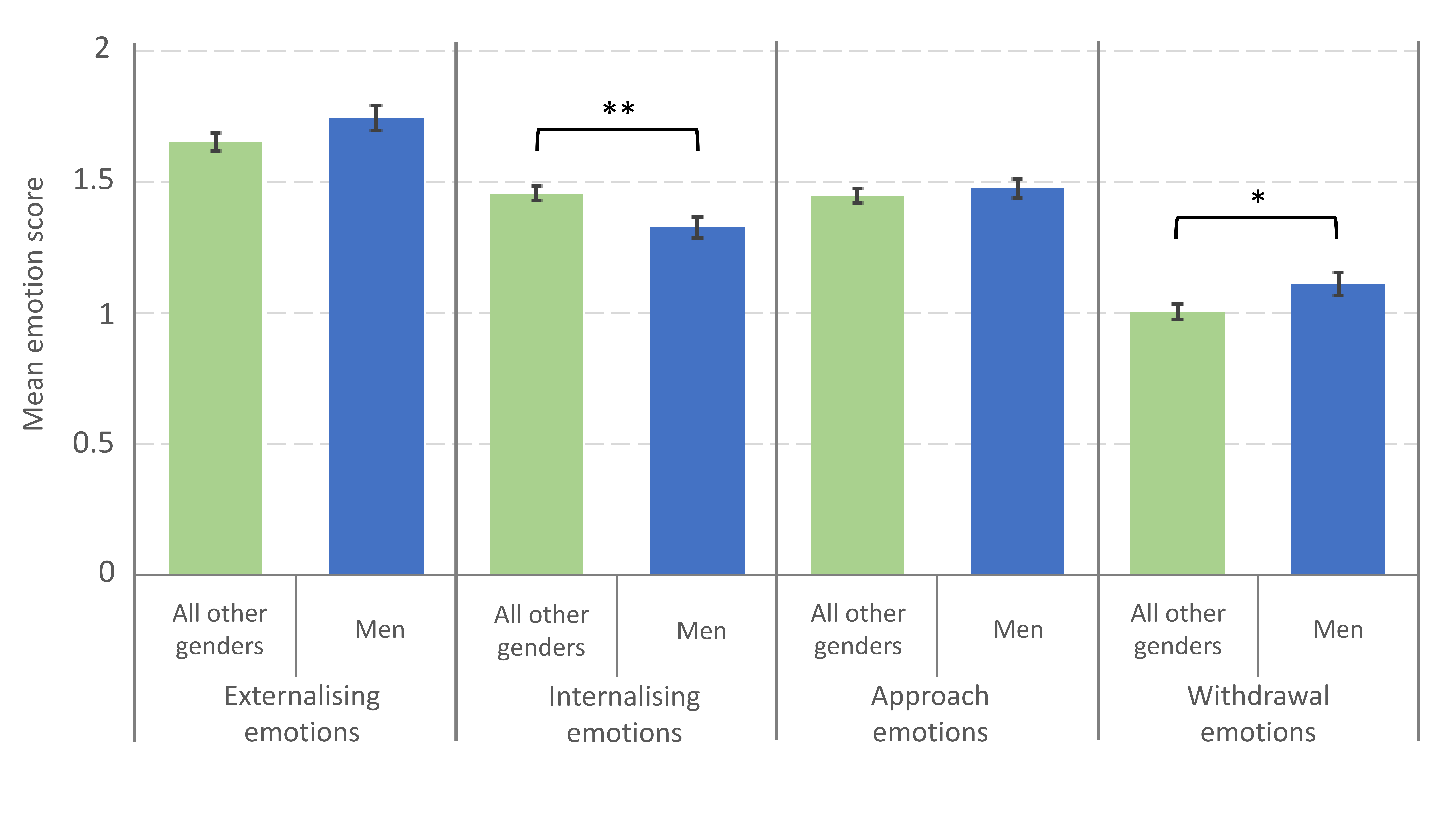

Supplement: S3 Fig — Differences between groups are presented at the mean covariate values: GAD-7 total score = 8.01, Climate distress total score = 13.0840. (TIF) [file pgph.0001938.s003.tif]
