# Supplementary material for: Psycho-social factors associated with climate distress, hope and behavioural intentions in young UK residents
Source: PLOS Glob Public Health. 2023 Aug 23;3(8):e0001938. doi: 10.1371/journal.pgph.0001938 (PMC10446227; doi:10.1371/journal.pgph.0001938)
Supplement: S1 Table — The full survey is available from: https://osf.io/9ewtn. (DOCX) [file pgph.0001938.s004.docx]

**Supplementary Information**

**S2 Table**

List of survey questions used in the current analyses. The full survey is available from: <https://osf.io/9ewtn>

| **Scale** | **Instructions** | **Questions/items** |
| --- | --- | --- |
| **PHQ9** | Thinking back to **the last 2 weeks**, could you tell us how often (if at all) you have felt bothered by the following things? | \|  \| Not at all \| Several days \| More than half the days \| Nearly every day \| \| --- \| --- \| --- \| --- \| --- \| \| Little interest or pleasure in doing things \|  \|  \|  \|  \| \| Feeling down, depressed, or hopeless \|  \|  \|  \|  \| \| Trouble falling asleep or staying asleep, or sleeping too much \|  \|  \|  \|  \| \| Feeling tired or having little energy \|  \|  \|  \|  \| \| Poor appetite or overeating \|  \|  \|  \|  \| \| Feeling bad about yourself- or that you’re a failure or have let yourself or your family down \|  \|  \|  \|  \| \| Trouble concentrating on things, such as reading or watching television \|  \|  \|  \|  \| \| Moving or speaking so slowly that other people could have noticed? Or the opposite- being so fidgety or restless that you have been moving around a lot more than usual \|  \|  \|  \|  \| \| Thoughts that you would be better off dead or hurting yourself in some way \|  \|  \|  \|  \| |
| **GAD-7** | Again, thinking back to the last 2 weeks, could you tell us how often (if at all) you have felt bothered by the following things? | \|  \| Not at all \| Several days \| More than half the days \| Nearly every day \| \| --- \| --- \| --- \| --- \| --- \| \| Feeling nervous, anxious or on edge \|  \|  \|  \|  \| \| Not being able to stop or control worrying \|  \|  \|  \|  \| \| Worrying too much about different things \|  \|  \|  \|  \| \| Trouble relaxing \|  \|  \|  \|  \| \| Being so restless that it is hard to sit still \|  \|  \|  \|  \| \| Becoming easily annoyed or irritable \|  \|  \|  \|  \| \| Feeling afraid as if something awful might happen \|  \|  \|  \|  \| |
| **PSS** | The following questions are about your feelings and thoughts **during the last month**. Please indicate how often you felt or thought a certain way. | \|  \| Never \| Almost never \| Sometimes \| Fairly often \| Very often \| \| --- \| --- \| --- \| --- \| --- \| --- \| \| Felt that you were unable to control the important things in your life \|  \|  \|  \|  \|  \| \| Felt confident about your ability to handle your personal problems \|  \|  \|  \|  \|  \| \| Felt that things were going your way \|  \|  \|  \|  \|  \| \| Felt difficulties were piling up so high that you could not overcome them \|  \|  \|  \|  \|  \| |
| **Climate change impacts** | Which events or changes associated with the climate change have had a negative effect on your life?  From the list below, please select up to three events/changes that have affected you the most. | - Seeing coverage in the media of the changes that are happening (loss of species, icecaps melting etc.) - Personal experience with effects of climate change (e.g. experienced natural disasters resulting from climate change such as floods, bush fires) - Family or friends have experienced the effects of climate change (e.g. experienced natural disasters resulting from climate change such as floods, bush fires) - Experiencing more extreme weather events where you live (e.g. severe heatwaves, heavier storms, more rain, ...) - Seeing natural environments that you care about change for the worse - Concerns for the health and safety of yourself and/or your loved ones - Worry, concern of frustration about others (people, companies) who do not support action against climate change - Increased worry about your own future prospects in life - Increased tension or agreements with family and/or loved ones - Feeling frustrated at your lack of control or opportunities to do something about climate change - Feeling guilty or ashamed about contributing to the problem of climate change through your own behaviours and choices |
|  | Overall, how much has climate change affected your life in a negative way? | - Not at all - A little - Moderately - A lot - Extremely |
|  | Which events or changes associated with the climate change have had a positive effect on your life?  From the list below, please select up to three events/changes that have affected you the most. | - Having more opportunities learn more about the natural world and how it changes - Feeling inspired to connect more with the natural world - Finding a like-minded community and solidarity through climate change (e.g. activism, learning) - Feeling inspired to live a more more eco-friendly life with improved health and wellbeing - Gaining a sense of purpose and/or identity by fighting against climate change - Enjoying more sunshine and higher temperatures in the UK - Feeling inspired and/or empowered by the actions of others to fight climate change - Seeing others change their behaviour as a result of my own actions against climate change |
|  | Overall, how much has climate change affected your life **in a positive way**? | - Not at all - A little - Moderately - A lot - Extremely |
| **Climate change emotions** | When you think about climate change, how strongly do you feel each of the following emotions (if at all)? | \|  \| Not at all \| A little \| Moderately \| Very much \| \| --- \| --- \| --- \| --- \| --- \| \| Interested \|  \|  \|  \|  \| \| Helpless \|  \|  \|  \|  \| \| Disgusted \|  \|  \|  \|  \| \| Afraid \|  \|  \|  \|  \| \| Outraged \|  \|  \|  \|  \| \| Angry \|  \|  \|  \|  \| \| Hopeful \|  \|  \|  \|  \| \| Ashamed \|  \|  \|  \|  \| \| Guilty \|  \|  \|  \|  \| \| Courageous \|  \|  \|  \|  \| \| Frustrated \|  \|  \|  \|  \| \| Disappointed \|  \|  \|  \|  \| \| Concerned \|  \|  \|  \|  \| \| Anxious \|  \|  \|  \|  \| \| Sad \|  \|  \|  \|  \| \| Disconnected/apathetic \|  \|  \|  \|  \| \| Engaged \|  \|  \|  \|  \| \| Isolated/lonely \|  \|  \|  \|  \| |
| **Climate Distress Scale** | How much do each of the following statements about the effect of the climate change apply to you? | \|  \| Does not describe my feelings \| Slightly describes my feelings \| Moderately describes my feelings \| Mostly describes my feelings \| Clearly describes my feelings \| \| --- \| --- \| --- \| --- \| --- \| --- \| \| I feel distressed each time I see or read media coverage of the impacts and consequences of climate change \|  \|  \|  \|  \|  \| \| At times I find myself thinking and worrying about what the world will really be like in the future because of climate change \|  \|  \|  \|  \|  \| \| I experience some guilt over the fact that my lifestyle and behaviour (e.g. consumption patterns) is in part responsible for the unfolding impacts of climate change \|  \|  \|  \|  \|  \| \| It upsets me that there seems to be so little that I can do to address environmental problems like climate change \|  \|  \|  \|  \|  \| \| At times I feel some personal responsibility for the problems and unfolding impacts of climate change \|  \|  \|  \|  \|  \| \| The threat of climate change is affecting my quality of life \|  \|  \|  \|  \|  \| \| The threat of climate change is affecting the way I think about environmental quality more generally \|  \|  \|  \|  \|  \| \| I feel some sense of loss because of climate change impacts that are becoming apparent in my local area \|  \|  \|  \|  \|  \| |
| **Climate activism** | Prior to the COVID-19 pandemic, were you involved in any **climate activism** (e.g. attending a march, participated in school strikes, petitions, fundraising, ...) | - No - Yes - please specify: ________________________________________________ |
| **Pro-environmental behaviour** | Prior to the COVID-19 pandemic, how often did you do things a certain way for environmental reasons?   This could include things such as recycling, taking public transport or your bike rather than driving, choosing a local holiday instead of flying somewhere, being vegan or vegetarian, limiting single-use plastics etc. | - Not at all - Occasionally - More often than not - All the time |
|  | Is there anything else you would like to share with us about your experience with climate change and the actions you have taken/continue to take to reduce the impact of climate change on your life and/or that of others? | Text box |
| **Hopes and dreams** | How often do you worry about how the following events/changes will affect your future? | \|  \| Never \| Seldom \| Sometimes \| Often \| Very often \| \| --- \| --- \| --- \| --- \| --- \| --- \| \| Climate change \|  \|  \|  \|  \|  \| \| My financial situation \|  \|  \|  \|  \|  \| \| Work or career \|  \|  \|  \|  \|  \| \| COVID-19 \|  \|  \|  \|  \|  \| \| School or studies \|  \|  \|  \|  \|  \| \| Personal relationships \|  \|  \|  \|  \|  \| \| Political issues and events \|  \|  \|  \|  \|  \| \| The economic situation \|  \|  \|  \|  \|  \| |
|  | Is there anything else you would like to share with us about your hopes for the future?   You can tell us about things you wish for, how you see this happening and possibly also share some things that you might fear or that you would like to avoid happening. | Text box |
