# Supplementary material for: Psycho-social factors associated with climate distress, hope and behavioural intentions in young UK residents
Source: PLOS Glob Public Health. 2023 Aug 23;3(8):e0001938. doi: 10.1371/journal.pgph.0001938 (PMC10446227; doi:10.1371/journal.pgph.0001938)
Supplement: S2 Table — (DOCX) [file pgph.0001938.s005.docx]

**Supplementary Information**

**S4 Table**

*Model results for the multinomial regression on distress categories; with inclusion of depression, anxiety and stress scores as predictors.*

|  | ***B*** | **(SE)** | **p-value** | **Odds ratio** | **95% CI Lower Bound** | **95% CI Upper Bound** |
| --- | --- | --- | --- | --- | --- | --- |
| Moderate distress^a^ | | | | | | |
| Intercept | -.668 | 1.019 | .512 |  |  |  |
| Age | -.062 | .041 | .131 | .940 | .867 | 1.019 |
| Gender = men | -.285 | .216 | .188 | .752 | .492 | 1.149 |
| Gender = women or other gender | 0^b^ | . | . | . | . | . |
| Cultural background/ethnicity = white/European | -.009 | .235 | .968 | .991 | .625 | 1.570 |
| Cultural background/ethnicity = any other | 0^b^ | . | . | . | . | . |
| Location = urban | .125 | .315 | .692 | 1.133 | .611 | 2.102 |
| Location = rural | 0^b^ | . | . | . | . | . |
| Socio-economic status ** | .136 | .048 | .005 | 1.145 | 1.042 | 1.259 |
| GAD-7 total score | .047 | .030 | .121 | 1.048 | .988 | 1.113 |
| PHQ-9 total score | .034 | .031 | .263 | 1.035 | .975 | 1.099 |
| PSS total score | .074 | .047 | .113 | 1.077 | .982 | 1.181 |
| High distress^a^ | | | | | | |
| Intercept | -1.862 | 1.669 | .264 |  |  |  |
| Age | -.088 | .067 | .190 | .915 | .802 | 1.045 |
| Gender = men ** | -1.260 | .437 | .004 | .284 | .121 | .668 |
| Gender = women or other gender | 0^b^ | . | . | . | . | . |
| Cultural background/ethnicity = white/European | .683 | .450 | .129 | 1.979 | .819 | 4.778 |
| Cultural background/ethnicity = any other | 0^b^ | . | . | . | . | . |
| Location = urban | -.627 | .463 | .176 | .534 | .215 | 1.324 |
| Location = rural | 0^b^ | . | . | . | . | . |
| Socio-economic status (FAS total score) | .054 | .079 | .494 | 1.056 | .904 | 1.233 |
| GAD-7 total score | .054 | .047 | .250 | 1.055 | .963 | 1.157 |
| PHQ-9 total score | .049 | .048 | .314 | 1.050 | .955 | 1.154 |
| PSS total score * | .170 | .081 | .036 | 1.186 | 1.011 | 1.390 |

*^a^ The reference category is low distress*

*^b^ This parameter is set to 0 because it is redundant*
