# Supplementary material for: Psycho-social factors associated with climate distress, hope and behavioural intentions in young UK residents
Source: PLOS Glob Public Health. 2023 Aug 23;3(8):e0001938. doi: 10.1371/journal.pgph.0001938 (PMC10446227; doi:10.1371/journal.pgph.0001938)
Supplement: S3 Table — ***p < .001; **p < .01. *p < .05 (applies to the omnibus test comparing all three levels of distress for presence/absence of each specific impact). In addition, we report follow-up pair-wise tests comparing low-moderate, moderate-high and low-high levels of distress groups on presence/absence of each of the specific impacts. (DOCX) [file pgph.0001938.s006.docx]

**Supplementary Information**

**S5 Table**

*Chi-square tests assessing the association between distress levels (low/moderate/high) and reporting (yes/no) of specific climate impacts. ***p<.001; **p<.01. *p<.05 (applies to the omnibus test comparing all three levels of distress for presence/absence of each specific impact). In addition, we report follow-up pair-wise tests comparing low-moderate, moderate-high and low-high levels of distress groups on presence*
