# Supplementary material for: Psycho-social factors associated with climate distress, hope and behavioural intentions in young UK residents
Source: PLOS Glob Public Health. 2023 Aug 23;3(8):e0001938. doi: 10.1371/journal.pgph.0001938 (PMC10446227; doi:10.1371/journal.pgph.0001938)
Supplement: S4 Table — (DOCX) [file pgph.0001938.s007.docx]

**Supplementary Information**

**S6 Table**

*Correlation matrix between climate distress, overall perceived extent of positive and negative climate impacts and climate-related emotions.*
