# Supplementary material for: Psycho-social factors associated with climate distress, hope and behavioural intentions in young UK residents
Source: PLOS Glob Public Health. 2023 Aug 23;3(8):e0001938. doi: 10.1371/journal.pgph.0001938 (PMC10446227; doi:10.1371/journal.pgph.0001938)
Supplement: S5 Table — (DOCX) [file pgph.0001938.s008.docx]

**Supplementary Information**

**S10 Table**

*Description and frequency of categories and sub-categories for the free-text responses pertaining to respondents’ reported “hopes for the future”.*

| **Categories and**  **Sub-categories** | | **Description** | **Example Quotes** | **Frequency of Codes** |
| --- | --- | --- | --- | --- |
| **COVID-related Factors** | |  |  | **N = 73** |
|  | Life to return to normal | Participants reported a desire for life to return to normal following the pandemic. This included hopes to find a vaccine. | *“I wish for things to be how they were before covid”*  *“Fear that the pandemic could be ongoing for a few years”* | n = 36  Hopeful = 83%  Fearful = 25% |
|  | Keep changes | Participants reported hopes that some of the changes brought about by the pandemic would remain in place. | *“I also hope this addition of hand sanitisers and hygiene procedures to public areas become the norm”*  *“I fear … everyone forgetting about their hygiene when in public”* | n = 14  Hopeful = 86%  Fearful = 14% |
|  | Lessons learned and future preparedness | Participants expressed hopes that lessons had been learned through the pandemic and that there would be greater preparedness for future situations. | *“Preparation of a structured plan for future pandemics”*  *“I fear that the government has purposely underprepared for a second COVID wave in winter”* | n = 23  Hopeful = 91%  Fearful = 9% |
| **Social Factors** | |  |  | **N = 53** |
|  | Reconnection and Community | Participants expressed hopes that they could reconnect with their loved ones and live in a caring community environment. | *“I want to see personal relationships get back to the way they were”*  *“I worry that it may be more difficult to make friends and form connections due to social distancing”* | n = 24  Hopeful = 92%  Fearful = 12% |
|  | Responsibility | Participants expressed hopes that individuals would take responsibility for each other and for future generations. This included companies and leaders being responsible for communities. | *“I hope people feel more accountable for their actions for the benefit of the wider community and future generations”*  *“I worry…the government not handling the situation properly, and people not following the rules”* | n = 19  Hopeful = 95%  Fearful = 5% |
|  | A good life and society | Participants expressed hopes for a general improvement in life, society, and the world. | *“I just want things to be better”*  *“A better society for all”*  *“We are heading for societal collapse”* | n = 10  Hopeful = 90%  Fearful = 10% |

| **Economic Factors** | |  |  | **N = 49** |
| --- | --- | --- | --- | --- |
|  | Economic recovery | Participants reported hopes for economic recovery following the pandemic. | *“I hope for the economy to recover quickly”*  *“I fear that the economic situation will lead to further austerity”* | n = 15  Hopeful = 73%  Fearful = 27% |
|  | Employment issues | Participants voiced concerns about employment issues and hopes for young people as a whole. | *“I hope that there are more jobs available”*  *“I fear an even more difficult job market”* | n = 17  Hopeful = 65%  Fearful = 35% |
|  | Cost of living | Participants discussed hopes about improvements in cost of living. This included costs associated with housing, transport, and education fees. | *“I hope to avoid an increase in price for everything…**I hope housing is affordable”*  *“I fear train ticket prices going up”* | n = 17  Hopeful = 47%  Fearful = 53% |
| **Policy Issues and Governance** | | | | **N=46** |
|  | Political change/action | Participants reported desires for political change in the UK and actions from world leaders. | *“I would really like to be led by a government that can show at least one ounce of empathy for the British Public, and who actually knows what it is like to live as a working-class individual”*  *“The UK Government isn’t helping”* | n = 18  Hopeful = 89%  Fearful = 11% |
|  | Justness and equality | Participants expressed hopes for just societal changes. This included hopes for greater equality in society, related to issues such as classism, racism, and discrimination against sexual minorities. | *“End of inequality between the rich getting richer and the poor getting poorer”*  *“I fear … the rich buying up vacant business lots on the cheap and receiving tax cuts while the rest of us go through another 10 years or more of austerity”* | n = 22  Hopeful = 86%  Fearful = 14% |
|  | Investment in amenities | Participants reported desire for investment in public amenities which would serve communities. | *“I wish for investment from municipalities in public spaces that are free and catered to community needs”*  *“I fear further cuts to public spending”* | n = 6  Hopeful = 67%  Fearful = 50% |
| **Environment and Climate** | | | | **N = 37** |
|  | Climate change action | Participants expressed hopes for climate change action. | *“I hope that world leaders will take the issue of climate change more seriously”*  *“I fear that there are people in positions of power who still don't believe in climate change”* | n = 22  Hopeful = 86%  Fearful = 14% |
|  | Other environmental factors | Participants hoped that the environment, and other living organisms, would be respected. | *“I hope more people felt connected to nature and realized how important it is to the planet and to our wellbeing”*  *“I fear that the government will not do enough to protect the natural environment in our country”* | n = 15  Hopeful = 93%  Fearful = 7% |
| **Personal Pursuits** | |  |  | **N = 27** |
|  | Employment and education goals | Participants expressed hopes in getting a job, obtaining a reasonable salary, or progressing their career. Participants also expressed goals around their education. | *“I hope to get a job sometime soon”*  *“I fear that because of the pandemic I will be unable to get a good job”* | n = 21  Hopeful = 81%  Fearful = 24% |
|  | Afford/buy a home | Participants expressed hopes to afford, buy or save for a house of their own. | *“I wish to buy my own house”* | n = 3  Hopeful = 100% |
|  | Hedonic wants/experiences | Participants discussed hedonic wants and experiences, such as being able to go to concerts or the pub like normal. | *“That I will be able to go out to a pub or bar and stay there past 10pm”* | n = 3  Hopeful = 100% |
| **Healthcare Factors** | |  |  | **N = 18** |
|  | Investment in health | Participants expressed hopes for greater investment in health systems and to avoid privatisation of the NHS. The importance of hygiene and the mental health sector was also highlighted. | *“I hope that the national health service can recover, and be better funded in future”*  *“I fear that our government will sell out the NHS and fully privatise health care”* | n = 14  Hopeful = 86%  Fearful = 14% |
|  | Improved pay and working conditions | Participants reported desires for improved pay and working conditions in healthcare. | *“Would like to see key workers be paid and respected more”* | n = 3  Hopeful = 100% |
|  | A good life and society | Participants expressed hopes for a general improvement in life, society, and the world. | *“I just want things to be better”*  *“A better society for all”*  *“We are heading for societal collapse”* | n = 10  Hopeful = 90%  Fearful = 10% |

***Note:*** *Different elements of a single free text response could be coded to represent multiple (sub-)categories, resulting in a higher count of specific category instances than the number of respondents (n=151). The total frequency for the sub-categories could also exceed the frequency of an overarching category because one free-text comment may have included information relevant to multiple sub-categories. Percentages may exceed 100% in some places as some participant responses included both hopeful and fearful framing.*
