# Supplementary material for: Psycho-social factors associated with climate distress, hope and behavioural intentions in young UK residents
Source: PLOS Glob Public Health. 2023 Aug 23;3(8):e0001938. doi: 10.1371/journal.pgph.0001938 (PMC10446227; doi:10.1371/journal.pgph.0001938)
Supplement: S6 Table — Note: B = regression coefficient, SE = standard error, Wald = Wald statistic, df = degrees of freedom, OR = odds ratio (defined as exp(b), CI = Confidence Interval. (DOCX) [file pgph.0001938.s009.docx]

**Supplementary Information**

**S11 Table**

*Results of the binary logistic regression model predicting participation in climate activism: model statistics at each step, model statistics for the comparison with the previous model (Δ Step), and individual predictor coefficients. Note: B=regression coefficient, SE=standard error, Wald = Wald statistic, df=degrees of freedom, OR=odds ratio (defined as exp(b), CI=Confidence Interval.*

|  | B | SE | Wald | df | p-value | OR | 95% CI for OR | |  |
| --- | --- | --- | --- | --- | --- | --- | --- | --- | --- |
|  |  |  |  |  |  |  | Lower | Upper |  |
| **Step 1: χ^2^(5)=21.752, p<.001** | | | | | | | | | |
| Gender (0=men; 1=other) | .477 | .253 | 3.569 | 1 | .059 | 1.611 | .982 | 2.643 |  |
| Age*** | -.147 | .043 | 11.463 | 1 | <.001 | .863 | .793 | .940 |  |
| Ethnicity/cultural background (0=white European; 1=other) | -.330 | .269 | 1.505 | 1 | .220 | .719 | .425 | 1.218 |  |
| Geographic location (0=urban; 1=rural) | .196 | .317 | .384 | 1 | .535 | 1.217 | .654 | 2.264 |  |
| Socio-economic status | .092 | .051 | 3.255 | 1 | .071 | 1.096 | .992 | 1.210 |  |
| Constant | .787 | 1.001 | .618 | 1 | .432 | 2.196 |  |  |  |
| **Δ Step 2: χ^2^(1)=39.964, p<.001; full model effects: χ^2^(6)=61.716, p<.001, Nagelkerke R^2^=.181** | | | | | | | | | |
| Gender (0=men; 1=other) | .183 | .266 | .473 | 1 | .492 | 1.201 | .713 | 2.023 |  |
| Age** | -.148 | .046 | 10.557 | 1 | .001 | .863 | .789 | .943 |  |
| Ethnicity/cultural background (0=white European; 1=other) | -.225 | .280 | .647 | 1 | .421 | .798 | .461 | 1.383 |  |
| Geographic location (0=urban; 1=rural) | .092 | .336 | .075 | 1 | .784 | 1.096 | .567 | 2.118 |  |
| Socio-economic status | .097 | .053 | 3.334 | 1 | .068 | 1.102 | .993 | 1.224 |  |
| Climate distress score*** | .116 | .020 | 34.939 | 1 | <.001 | 1.123 | 1.081 | 1.167 |  |
| Constant | -.726 | 1.075 | .456 | 1 | .499 | .484 |  |  |  |
| **Δ Step 3: χ^2^(4)=64.852, p<.001,** **full model effects: χ^2^(10)=126.568, p<.001, Nagelkerke R^2^=.349** | | | | | | | | | |
| Gender (0=men; 1=other) | .320 | .302 | 1.118 | 1 | .290 | 1.377 | .761 | 2.490 |  |
| Age** | -.162 | .050 | 10.670 | 1 | .001 | .850 | .771 | .937 |  |
| Ethnicity/cultural background (0=white European; 1=other) | -.259 | .311 | .695 | 1 | .404 | .772 | .420 | 1.419 |  |
| Geographic location (0=urban; 1=rural) | .254 | .365 | .485 | 1 | .486 | 1.290 | .630 | 2.640 |  |
| Socio-economic status | .057 | .059 | .944 | 1 | .331 | 1.059 | .943 | 1.189 |  |
| Climate distress score* | .072 | .030 | 5.961 | 1 | .015 | 1.075 | 1.014 | 1.140 |  |
| Externalising emotions*** | .871 | .196 | 19.662 | 1 | <.001 | 2.388 | 1.626 | 3.510 |  |
| Internalising emotions | -.246 | .215 | 1.316 | 1 | .251 | .782 | .513 | 1.191 |  |
| Approach emotions*** | .719 | .182 | 15.561 | 1 | <.001 | 2.052 | 1.436 | 2.933 |  |
| Withdrawal emotions** | -.478 | .164 | 8.469 | 1 | .004 | .620 | .450 | .856 |  |
| Constant | -1.630 | 1.197 | 1.854 | 1 | .173 | .196 |  |  |  |
