# Supplementary material for: Psycho-social factors associated with climate distress, hope and behavioural intentions in young UK residents
Source: PLOS Glob Public Health. 2023 Aug 23;3(8):e0001938. doi: 10.1371/journal.pgph.0001938 (PMC10446227; doi:10.1371/journal.pgph.0001938)
Supplement: S7 Table — Note: B = regression coefficient, SE = standard error, Wald = Wald statistic, df = degrees of freedom, OR = odds ratio (defined as exp(B), CI = Confidence Interval, AIC = Akaike Information Criterion. (DOCX) [file pgph.0001938.s010.docx]

**Supplementary Information**

**S12 Table**

*Results of the ordinal regression models predicting self-reported participation in pro-environmental behaviours: model statistics at each step, model fit, and individual predictor coefficients. Note: B=regression coefficient, SE=standard error, Wald = Wald statistic, df=degrees of freedom, OR=odds ratio (defined as exp(B), CI=Confidence Interval, AIC=Akaike Information Criterion.*

|  | B | SE | Wald | df | p-value | 95% CI for OR | |
| --- | --- | --- | --- | --- | --- | --- | --- |
|  |  |  |  |  |  | Lower bound | Upper bound |
| **Step 1: χ^2^(5)=14.838, p=.011, AIC=854.182** | | | | | | | |
| Gender (0=men; 1=other) | .039 | .1927 | .040 | 1 | .841 | -.339 | .416 |
| Age | .051 | .0356 | 2.044 | 1 | .153 | -.019 | .121 |
| Ethnicity/cultural background (0=white European; 1=other)* | .445 | .2057 | 4.669 | 1 | .031 | .041 | .848 |
| Geographic location (0=urban; 1=rural) | .005 | .2717 | .000 | 1 | .984 | -.527 | .538 |
| Socio-economic status* | .093 | .0414 | 4.998 | 1 | .025 | .011 | .174 |
| **Step 2: χ^2^(6)=66.416, p<.001, AIC=804.604** | | | | | | | |
| Gender (0=men; 1=other) | .324 | .2033 | 2.537 | 1 | .111 | -.075 | .722 |
| Age | .072 | .0370 | 3.770 | 1 | .052 | -.001 | .144 |
| Ethnicity/cultural background (0=white European; 1=other) | .396 | .2123 | 3.488 | 1 | .062 | -.020 | .813 |
| Geographic location (0=urban; 1=rural) | .083 | .2829 | .087 | 1 | .768 | -.471 | .638 |
| Socio-economic status* | .084 | .0428 | 3.871 | 1 | .049 | .000 | .168 |
| Climate distress score*** | .109 | .0160 | 46.753 | 1 | <.001 | .078 | .141 |
| **Step 3: χ^2^(10)=93.327, p<.001, AIC=785.694** | | | | | | | |
| Gender (0=men; 1=other) | .350 | .2100 | 2.777 | 1 | .096 | -.062 | .761 |
| Age | .071 | .0377 | 3.497 | 1 | .061 | -.003 | .145 |
| Ethnicity/cultural background (0=white European; 1=other)* | .512 | .2226 | 5.285 | 1 | .022 | .075 | .948 |
| Geographic location (0=urban; 1=rural) | .059 | .2849 | .042 | 1 | .837 | -.500 | .617 |
| Socio-economic status | .067 | .0435 | 2.405 | 1 | .121 | -.018 | .153 |
| Climate distress score*** | .084 | .0227 | 13.558 | 1 | <.001 | .039 | .128 |
| Externalising emotions | .156 | .1477 | 1.112 | 1 | .292 | -.134 | .445 |
| Internalising emotions | -.061 | .1651 | .137 | 1 | .711 | -.385 | .263 |
| Approach emotions*** | .615 | .1413 | 18.944 | 1 | <.001 | .338 | .892 |
| Withdrawal emotions | -.107 | .1237 | .752 | 1 | .386 | -.350 | .135 |
