# Supplementary material for: Psycho-social factors associated with climate distress, hope and behavioural intentions in young UK residents
Source: PLOS Glob Public Health. 2023 Aug 23;3(8):e0001938. doi: 10.1371/journal.pgph.0001938 (PMC10446227; doi:10.1371/journal.pgph.0001938)
Supplement: S8 Table — (DOCX) [file pgph.0001938.s011.docx]

**Supplementary Information**

**S13 Table**

*Description and frequency of categories and sub-categories for the free-text responses pertaining to “Climate change experiences and actions”.*

| **Categories and Sub-Categories** | **Description** | **Example Quotes** | **Frequency of codes** |
| --- | --- | --- | --- |
| **Individual Actions N=94** | | | |
| Private sphere behaviours | Describes or gives examples of specific actions they have taken in their own lives. | *“My friends and I regularly cycle to see each other instead of taking an Uber”.* | N=60 |
| Exerting social influence | Describes how they have attempted to or influenced people around them to take up certain behaviours. | *“I convinced my parents to install solar panels on their house.”* | N=13 |
| Civic participation/activism | Considers the need for collective action as a way to combat climate change, including influencing political decision making/lobbying for policy changes. | *“I hope people will use their vote to encourage more climate action.”* | N=6 |
| Aspirations | Expresses a desire or plan to take certain actions, or a broad, non-specific ambition be more involved in pro-environmental or pro-climate actions. | *“I have become more aware of my own impact and my hope is to buy an electric vehicle sometime soon.”* | N=4 |
| Reflections on  (motivations for) action | Comments on the reasons or consequences of taking action, and/or describes the values that underpin the need to act on climate change. | *“I became a vegetarian for animal welfare reasons, but I am now also very convinced that it is much better for the planet.”* | N=11 |
| **Barriers to Individual Action N=76** | | | |
| Lack of efficacy | Expresses the belief that individual actions are have little impact in the grand scheme of things. | *“**While I do some things […] I just don’t think it makes much difference in the grand scheme of it all.”* | N=25 |
| Contextual factors | Describes practical or situational barriers, or lack of control, that get in the way of engaging with climate action or pro-environmental behaviour. | *“I’d love to go zero-waste, but there aren’t many shops around where I live, and it soon becomes prohibitively expensive.”* | N=12 |
| Deferring of responsibility | Questions whether the individual should be held responsible, lays the blame elsewhere, or identifies specific other actors who are assumed to be more responsible for taking action (e.g., corporates or governments). | *“It’s the fat cat corporations that really should be making changes.”* | N=33 |
| Lack of concern/priority | The observation or belief that (generally other) people are too busy or have other concerns to worry about first. | *“Honestly, I don’t think most people care it about it too much, given other problems like COVID and the economy.”* | N=5 |
| Lack of awareness | Expresses the belief that (other) people may not have sufficient knowledge about what to do about climate change or what pro-environmental actions they could take. | *“I wish more people were aware of their impact. My parents’ generation just don’t see to be on top of this like we are?”* | N=1 |
| **Climate Change Knowledge and Beliefs N=26** | | | |
| Impact and efficacy beliefs | Reflects on whether or not climate change can be halted or reversed or expresses the belief that something can (still) be done (or not). | *“I don’t think it’s too late to act, but we have to change things now.”* | N=20 |
| Recognition of fact | Declares knowledge around climate change and the impacts, or expresses the belief that climate change is real, is occurring in the here and now or that the impacts are being felt. | *“It’s obvious that climate change is happening – just think of the wildfires and increasing number of wild weather events.”* | N=6 |
| **System-Level Barriers and Levers N=18** | | | |
| Government commitment and support | Specifically points to failures of government to support climate action, or identifies (future) opportunities, specific or general, for government intervention. | *“The Tory government just doesn’t see climate change as a priority.”* | N=8 |
| Economic/capitalist system pressures | Points to economic drivers (e.g., greed, consumerism) that stand in the way of achieving climate goals. Also includes expressions of the need for a large-scale (global) overhaul, or systemic changes. | *“We need to shift away from consumerism and materialist cultures and think about distributing power and wealth.”* | N=8 |
| Alternative technologies | Points to large-scale technological, logistical, operational barriers or identifies potential (new) technological solutions for climate mitigation or adaptation. | *“Carbon capture and storage might work, but I don’t know if we’re ready for that.”* | N=2 |
| **Psychological Factors N=9** | | | |
| Emotional impacts | Expresses specific feelings that the respondent has experienced in relation to climate change, including positive (e.g., feeling hopeful) and negative ones (e.g., feeling sad or defeated). | *“I try to do my part, but I also feel guilty because I could be doing more.”* | N=7 |
| Coping strategies | Describes actions they take to deal with or process their feelings around climate change. | *“I try not to think about it too much, so sometimes I just watch stupid things on the tv and never really watch the news”.* | N=2 |

**Note:** Different elements of a single free text response could be coded to represent multiple (sub-)categories, resulting in a higher count of specific category instances than the number of respondents (n=121). The total frequency for the sub-categories could also exceed the frequency of an overarching category because one free-text comment may have included information relevant to multiple sub-categories.
