# Supplementary material for: Psycho-social factors associated with climate distress, hope and behavioural intentions in young UK residents
Source: PLOS Glob Public Health. 2023 Aug 23;3(8):e0001938. doi: 10.1371/journal.pgph.0001938 (PMC10446227; doi:10.1371/journal.pgph.0001938)

**Supplementary Information**

**S1 Text**

*Example adverts used to recruit participants on social media.*

“Aged 16-24 and live in the UK?

Take part in our new @imperialcollege study to help us understand how our changing world makes you feel

Opportunity to win High Street shopping vouchers worth up to £50

Go to bit.ly/2Pj5m9B to take the survey. Please RT”


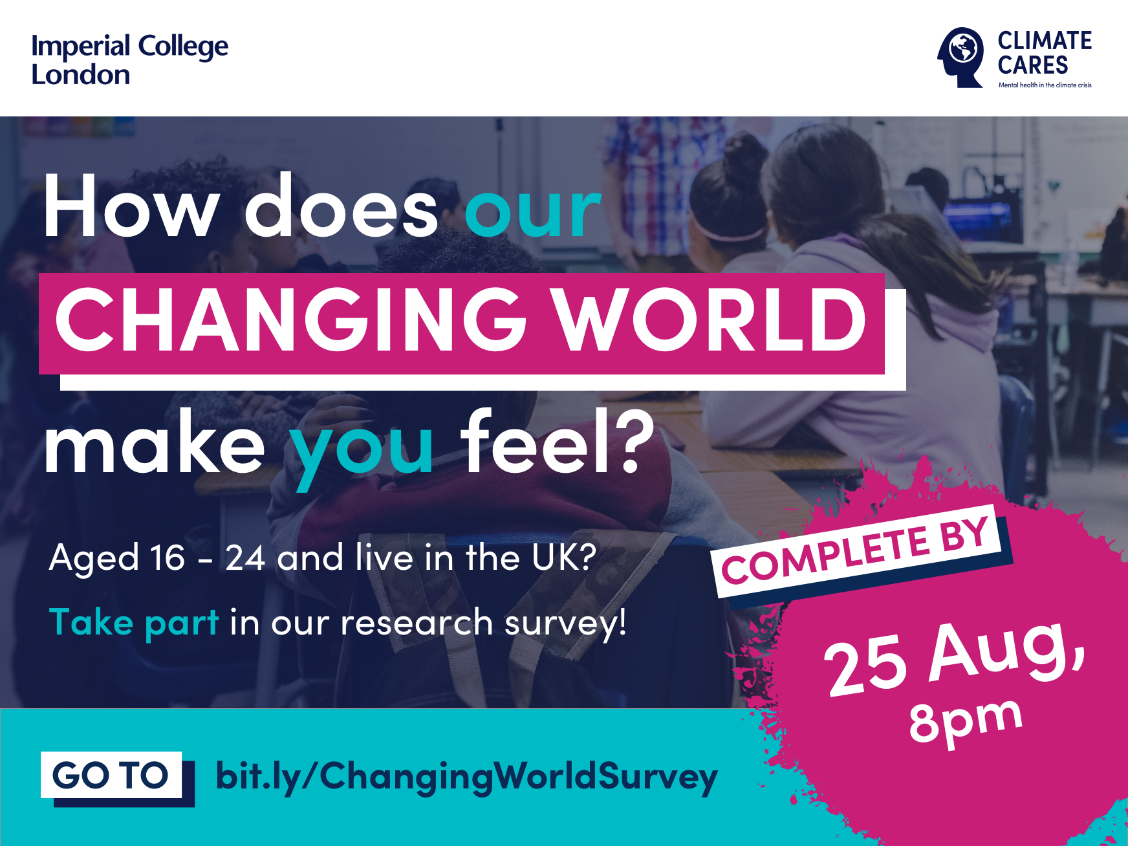

Supplement: S1 Text — (DOCX) [file pgph.0001938.s012.docx]
