# Supplementary material for: Psycho-social factors associated with climate distress, hope and behavioural intentions in young UK residents
Source: PLOS Glob Public Health. 2023 Aug 23;3(8):e0001938. doi: 10.1371/journal.pgph.0001938 (PMC10446227; doi:10.1371/journal.pgph.0001938)
Supplement: S2 Text — (DOCX) [file pgph.0001938.s013.docx]

**Supplementary Information**

**S9 Text**

*Statistical tests comparing reported emotional responses to climate change between genders (men vs other genders)*

Young men (n=172) reported different patterns of emotional responses to climate change compared to women and non-binary individuals (n=340). We conducted a multivariate GLM with scores on the four emotion categories as the dependent variables, and gender as the main factor. To control for differences in overall climate distress and generalised anxiety, Climate Distress Scale scores and GAD-7 scores were included as covariates. We observed a significant main effect of gender (F(4,505)=7.021, p<.001, $\eta_{p}^{2}$=.053), after controlling for the covariate effects, which was significant for the GAD-7 score (F(4,505)=19.714, p<.001, $\eta_{p}^{2}$=.135) and the climate distress score (F(4,503)=172.544, p<.001, $\eta_{p}^{2}$=.577. Univariate analyses revealed a significant gender difference with men less likely than other genders to report ‘internalising emotions’ (F(1,508)=6.872, p=.009, $\eta_{p}^{2}$=.013), but more likely to report ‘withdrawal’ emotions (F(1,508)=4.839, p=.028, $\eta_{p}^{2}$=.009). No significant gender differences were found for ‘externalising’ (F(1,508)=2.389, p=.123, $\eta_{p}^{2}$=.005) or ‘approach’ emotions (F(1,508)=0.287, p=.529, $\eta_{p}^{2}$=.001).
